# Could Less Be More? Accounting for Fractional-Dose Regimens and Different Number of Vaccine Doses When Measuring the Impact of the RTS,S/AS01_E_ Malaria Vaccine

**DOI:** 10.1093/infdis/jiae075

**Published:** 2024-03-04

**Authors:** Nelli Westercamp, Lawrence Osei-Tutu, Lode Schuerman, Simon K Kariuki, Anne Bollaerts, Cynthia K Lee, Aaron M Samuels, Christian Ockenhouse, Dennis K Bii, Samuel Adjei, Martina Oneko, Marc Lievens, Maame Anima Attobrah Sarfo, Cecilia Atieno, Ashura Bakari, Tony Sang, Maame Fremah Kotoh-Mortty, Kephas Otieno, François Roman, Patrick Boakye Yiadom Buabeng, Yaw Ntiamoah, Daniel Ansong, Tsiri Agbenyega, Opokua Ofori-Anyinam

**Affiliations:** Malaria Branch, Division of Parasitic Diseases and Malaria, Global Health Center, Centers for Disease Control and Prevention, Atlanta, Georgia, USA; Kwame Nkrumah University of Science and Technology, Agogo Presbyterian Hospital, Asante Akyem, Ghana; GSK, Wavre, Belgium; Centre for Global Health Research, Kenya Medical Research Institute, Kisumu, Kenya; GSK, Wavre, Belgium; Center for Vaccine Innovation and Access, PATH's Malaria Vaccine Initiative, Washington, DC, USA; Malaria Branch, Division of Parasitic Diseases and Malaria, Global Health Center, Centers for Disease Control and Prevention, Atlanta, Georgia, USA; Malaria Branch, Division of Parasitic Diseases and Malaria, Global Health Center, Centers for Disease Control and Prevention, Kisumu, Kenya; Center for Vaccine Innovation and Access, PATH's Malaria Vaccine Initiative, Washington, DC, USA; Centre for Global Health Research, Kenya Medical Research Institute, Kisumu, Kenya; Kwame Nkrumah University of Science and Technology, Agogo Presbyterian Hospital, Asante Akyem, Ghana; Centre for Global Health Research, Kenya Medical Research Institute, Kisumu, Kenya; GSK, Wavre, Belgium; Kwame Nkrumah University of Science and Technology, Agogo Presbyterian Hospital, Asante Akyem, Ghana; Centre for Global Health Research, Kenya Medical Research Institute, Kisumu, Kenya; Kwame Nkrumah University of Science and Technology, Agogo Presbyterian Hospital, Asante Akyem, Ghana; Centre for Global Health Research, Kenya Medical Research Institute, Kisumu, Kenya; Kwame Nkrumah University of Science and Technology, Agogo Presbyterian Hospital, Asante Akyem, Ghana; Centre for Global Health Research, Kenya Medical Research Institute, Kisumu, Kenya; GSK, Wavre, Belgium; Kwame Nkrumah University of Science and Technology, Agogo Presbyterian Hospital, Asante Akyem, Ghana; Kwame Nkrumah University of Science and Technology, Agogo Presbyterian Hospital, Asante Akyem, Ghana; Kwame Nkrumah University of Science and Technology, Agogo Presbyterian Hospital, Asante Akyem, Ghana; Kwame Nkrumah University of Science and Technology, Agogo Presbyterian Hospital, Asante Akyem, Ghana; GSK, Wavre, Belgium

**Keywords:** fractional-dose regimen, malaria vaccine, RTS, S/AS01_E_, vaccine efficacy, vaccine impact

## Abstract

**Background:**

The RTS,S/AS01_E_ (RTS,S) malaria vaccine is recommended for children in malaria endemic areas. This phase 2b trial evaluates RTS,S fractional- and full-dose regimens in Ghana and Kenya.

**Methods:**

In total, 1500 children aged 5–17 months were randomized (1:1:1:1:1) to receive RTS,S or rabies control vaccine. RTS,S groups received 2 full RTS,S doses at months 0 and 1 and either full (groups R012-20, R012-14-26) or fractional doses (one-fifth; groups Fx012-14-26, Fx017-20-32).

**Results:**

At month 32 post-dose 1, vaccine efficacy against clinical malaria (all episodes) ranged from 38% (R012-20; 95% confidence interval [CI]: 24%–49%) to 53% (R012-14-26; 95% CI: 42%–62%). Vaccine impact (cumulative number of cases averted/1000 children vaccinated) was 1344 (R012-20), 2450 (R012-14-26), 2273 (Fx012-14-26), and 2112 (Fx017-20-32). To account for differences in vaccine volume (fractional vs full dose; post hoc analysis), we estimated cases averted/1000 RTS,S full-dose equivalents: 336 (R012-20), 490 (R012-14-26), 874 (Fx012-14-26), and 880 (Fx017-20-32).

**Conclusions:**

Vaccine efficacy was similar across RTS,S groups. Vaccine impact accounting for full-dose equivalence suggests that using fractional-dose regimens could be a viable dose-sparing strategy. If maintained through trial end, these observations underscore the means to reduce cost per regimen thus maximizing impact and optimizing supply.

**Clinical Trials Registration:**

NCT03276962 (ClinicalTrials.gov).

## Teaser

Full- and fractional-dose regimens of the RTS,S/AS01_E_ malaria vaccine have similar vaccine efficacy against clinical malaria. With a fixed supply of vaccine, more children can be vaccinated and thus more cases prevented by using fractional- rather than full-dose regimens.

The RTS,S/AS01_E_ (RTS,S) malaria vaccine is currently the only malaria vaccine recommended for use in children from areas with moderate to high risk of malaria. We are conducting a trial in children in Ghana and Kenya to compare regimens with the third and subsequent RTS,S doses reduced to one-fifth and/or delayed vs the standard regimen, full-dose regimens, and a control vaccine. While we did not demonstrate superior vaccine efficacy of fractional- vs full-dose regimens, we showed that all RTS,S regimens provided substantial protection against malaria and were immunogenic and well tolerated during 32 months from the first RTS,S dose. We also calculated vaccine impact, measured as the number of malaria cases prevented for each 1000 children vaccinated with RTS,S vs the control vaccine. Up to study month 32, we found no systematic differences between full- and fractional-dose RTS,S regimens. However, children receiving fractional doses receive less vaccine. Therefore, we also estimated the number of malaria cases prevented by 1000 RTS,S full doses. We found that fractional-dose regimens can prevent a higher number of cases for the same volume of vaccine administered. These findings suggest that fractional RTS,S doses could be used to improve the impact of the malaria vaccine program with the available vaccine supply.

Despite being a preventable and treatable disease, malaria continues to cause significant morbidity and mortality in children aged <5 years, especially in sub-Saharan Africa [1]. Vaccines RTS,S/AS01_E_ (RTS,S) [2] and R21 [3] are recommended by the World Health Organization (WHO) for widespread use for children living in areas with moderate to high *Plasmodium falciparum* malaria transmission. The WHO recommends the administration of RTS,S according to a 4-dose schedule from 5 months of age, with the first 3 doses administered at least 4 weeks apart. The fourth dose can be administered 12 to 18 months after dose 3, which offers flexibility in dosing for efficient delivery.

Additionally, seasonally timed 5-dose schedules can be considered in areas with high seasonal or perennial malaria transmission with regular peaks. This strategy maximizes vaccine impact by overlapping the period of maximum vaccine efficacy (VE) and that of the highest malaria transmission [2]. RTS,S vaccination in combination with seasonal malaria chemoprevention (SMC) in children aged 5 to 17 months was shown to provide superior protection against uncomplicated clinical malaria in seasonal settings in Burkina Faso and Mali; the protective efficacy of the RTS,S + SMC combination was 71.7% and 58.6% over 1 and 3 years of follow-up, respectively, as compared with SMC alone [4].

Demand for malaria vaccines is expected to increase in the near future [2]. Limited vaccine supplies have prompted WHO recommendations for the use of fractional doses for vaccine-preventable diseases such as yellow fever, rabies, and poliomyelitis [5–7]. A similar approach could be considered to mitigate potential short-term RTS,S vaccine stockouts and increase its cost-effectiveness in the long term, thus enhancing the potential impact on sustained access to lifesaving vaccinations.

Previous controlled human malaria infection studies [8–10] suggested an improved VE for delayed fractional-dose RTS,S regimens. We conducted a phase 2b trial to assess whether this finding is confirmed in children in natural exposure settings. To this end, we evaluated different fractional-dose RTS,S regimens in children from Ghana and Kenya, as compared with a control group receiving rabies vaccine. The primary objective was not met: to demonstrate superior VE of a regimen with a third fractional dose (Fx012) vs the standard full-dose regimen (R012) over 12 months after dose 3 (at month 20 [M20]). However, all fractional- and full-dose RTS,S regimens provided substantial similar protection against clinical malaria when compared with the control group over 20 months of follow-up. VE estimates against clinical malaria ranged from 34% to 54%, and 701 to 1796 cases of clinical malaria per 1000 vaccinated children were averted across RTS,S groups [11]. These results provide the potential for flexibility in dosing regimen and schedule.

Here, we present trial results up to 33 months since first vaccination (M33), including VE and vaccine impact (at M32), immunogenicity, and safety (at M33) after administration of up to 5 RTS,S doses. To further quantify the potential additional benefit of fractional-dose regimens, we conducted a post hoc analysis of vaccine impact expressed as cases averted per 1000 RTS,S full-dose equivalents administered.

## METHODS

### Study Design and Objectives

The study design has been described in detail [11] and is summarized in Supplementary Figure 1. Briefly, we conducted an open-label, phase 2b, randomized controlled trial at the Malaria Research Center, Agogo, Ashanti Region (Ghana), and the Kenya Medical Research Institute and the US Centers for Disease Control and Prevention site in Siaya County (Kenya). We enrolled children aged 5 to 17 months and randomly assigned them to 1 of 5 groups (1:1:1:1:1): control (receiving rabies vaccine according to a M012 schedule) or 1 of 4 groups to receive 2 full doses of RTS,S at M0 and M1, followed by full doses at M2 and M20 (group R012-20 [standard regimen]); full doses at M2, M14, M26, and M38 (group R012-14-26); fractional doses at M2, M14, M26, and M38 (group Fx012-14-26); or fractional doses at M7, M20, and M32 (group Fx017-20-32). Fractional doses were administered as one-fifth (0.1 mL) of the full RTS,S vaccine volume (0.5 mL) after reconstitution.

We report VE and vaccine impact against clinical malaria (all episodes) meeting the primary and secondary case definitions up to M32—specifically, 12 months after dose 4 (groups R012-20 and Fx017-20-32) or 6 months after dose 5 (groups R012-14-26 and Fx012-14-26). We also present immune responses against circumsporozoite (CS) protein and hepatitis B surface antigen and safety data up to M33, when children in all RTS,S groups had received at least 4 vaccine doses (see Supplementary Table 1 for other objectives).

The protocol of the trial (ClinicalTrials.gov NCT03276962) was approved by relevant authorities, institutional review boards, and independent ethics committees. The study was overseen by an independent data monitoring committee and conducted according to the principles of the Declaration of Helsinki. Parents/guardians of all children signed an informed consent form.

### Procedures

Study procedures were previously described in detail [11]. For analyses presented here, malaria cases were detected via passive detection (ie, parents/guardians were encouraged to bring their children to the study-designated health care facility in case of illness). The primary case definition for clinical malaria was *P falciparum* asexual parasitemia >5000 parasites/μL and fever (axillary temperature ≥37.5 °C) at presentation [12]. The secondary case definition was *P falciparum* asexual parasitemia >0 parasites/μL and fever at presentation or history of fever within 24 hours of presentation. Parasitemia was determined by microscopy.

Blood samples for immunogenicity assessments were collected as indicated in Supplementary Figure 1 and analyzed as previously described [11, 13].

We present the following herein: solicited local and general adverse events (AEs) within 4 days after dose 5 and unsolicited AEs occurring within 30 days after any vaccination, cases of severe and cerebral malaria, serious AEs (SAEs), AEs of specific interest (seizures within 30 days postvaccination, meningitis, potential immune-mediated diseases), and AEs leading to withdrawal up to M33.

### Statistical Analysis

Sample size considerations were previously presented [11].

VE estimates (RTS,S vs control) against all episodes of clinical malaria were calculated with 95% CIs as 100 × (1 – incidence rate ratio), overall (adjusted for country as a fixed effect), and by country. Furthermore, they were analyzed by negative binomial regression allowing for interdependence between episodes within the same child; *P* values were calculated from the same model [14]. Incremental VE was estimated by comparing a fractional-dose regimen with a full-dose regimen.

We estimated vaccine impact as the number of cases averted over the relevant period per 1000 children vaccinated. To account for the actual volume of vaccine administered (ie, a fractional RTS,S dose corresponds to 0.2 full-dose equivalents), in a post hoc analysis we estimated vaccine impact for each RTS,S group as the number of clinical malaria cases averted per 1000 full doses administered (equation 1).


(1) $$\begin{array}{rl} & \begin{array}{c} \mathrm{Vaccine}\;\mathrm{impact}\;\mathrm{over}\;\mathrm{the}\\ \mathrm{considered}\;\mathrm{interval}\\ \mathrm{per}\;1000\;\mathrm{RTS},S\\ \mathrm{full}\text{-}\mathrm{dose}\;\mathrm{equivalents}\\ \mathrm{administered} \end{array}=\frac{\begin{array}{c} \mathrm{Number}\;\;\mathrm{of}\;\;\mathrm{clinical}\;\;\mathrm{cases}\;\;\\ \mathrm{averted}\;\;\mathrm{over}\;\;\mathrm{the}\;\;\mathrm{considered}\;\\ \mathrm{interval}/1000\;\;\mathrm{children}\;\;\mathrm{vaccinated} \end{array}}{\sum_{i=1}^{n}\;D_{i}} \end{array}$$

where *D_i_* = RTS,S full-dose equivalent for each vaccine dose planned per protocol in the considered interval (M0–M32) and RTS,S group (groups R012-20 and Fx017-20-32, n = 4; groups R012-14-26 and Fx012-14-26, n = 5).

VE/vaccine impact analyses were carried out on the exposed set (including children who received ≥1 vaccination), unless otherwise specified. Immunogenicity and reactogenicity analyses were conducted in a subset of 250 children; all other safety analyses were conducted in the exposed set. The percentage of doses followed by at least 1 unsolicited AE or any SAE within 30 days postvaccination was computed with 95% confidence intervals (CIs; post hoc analysis).

All analyses were performed with SAS version 9.4 (SAS Institute Inc).

## RESULTS

In total, 1609 children were randomized [11] and 1500 were included in the exposed set (Supplementary Figure 2). Baseline characteristics for the exposed set were similar across groups and countries (Supplementary Table 2).

**Figure 1.**
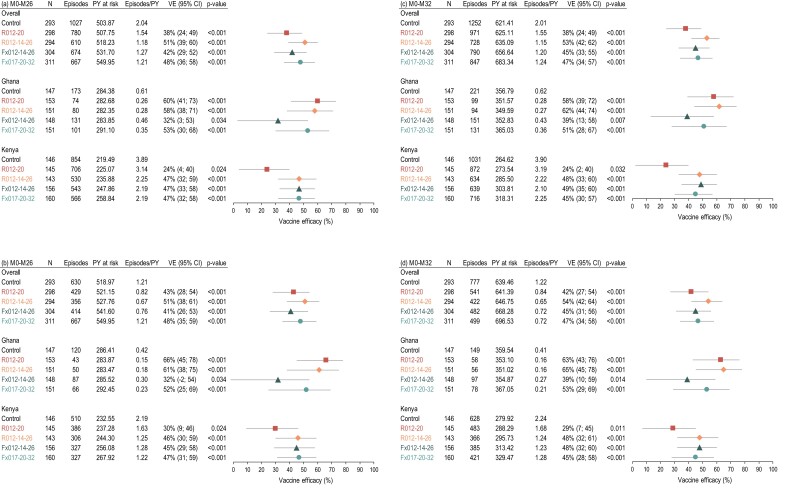
Vaccine efficacy against all episodes of clinical malaria meeting the secondary (*A*, *C*) and primary (*B*, *D*) case definitions up to study months 26 (*A*, *B*) and 32 (*C*, *D*), overall and by country (exposed set). Error bars represent 95% CIs. The trial was not powered to assess vaccine efficacy by country. M, month; N, number of children; PY, person-years; VE, vaccine efficacy; CI, confidence interval.

VE was in the same range in all groups, regardless of the case definition (Figure 1; Supplementary Table 3). VE against all episodes of clinical malaria varied from 38% (95% CI, 24%–49%) to 51% (95% CI, 39%–60%) for the secondary case definition and 41% (95% CI, 26%–53%) to 51% (95% CI, 38%–61%) for the primary case definition over 26 months from first dose (M0–M26), at 6 months after dose 4 in groups R012-20 and Fx017-20-32, and at 12 months after dose 4 in groups R012-14-26 and Fx012-14-26 (Figure 1*A* and 1*B*). Over 32 months from the first dose (M0–M32), at 12 months after dose 4 in groups R012-20 and Fx017-20-32, and at 6 months after dose 5 in groups R012-14-26 and Fx012-14-26, VE ranged from 38% (95% CI, 24%–49%) to 53% (95% CI, 42%–62%) for the secondary case definition and 42% (95% CI, 27%–54%) to 54% (95% CI, 42%–64%) for the primary case definition (Figure 1*C* and 1*D*). Overall, VE point estimates were higher for Ghana than Kenya for all endpoints assessed and in all groups except for Fx012-14-26 (Figure 1, Supplementary Table 4). Consistent with the previously reported M20 data set [11], the incremental VE of fractional- vs full-dose regimens did not indicate a substantial difference between regimens and was not statistically significant at M26 and M32 (Table 1, Supplementary Table 5).

**Table 1.** Incremental Efficacy of a Fractional Third Dose Administered at M2 Against All Clinical Malaria Episodes Meeting the Secondary Case Definition to M26 and M32: Overall (Exposed Set)

Period: Group	No.	Episodes	PY at Risk	Episodes/PY	IVE (95% CI), %	*P* Value
M0–M26						
R012-20	298	780	507.75	1.54	…	…
Fx012-14-26	304	674	531.70	1.27	7 (−17 to 26)	.529
R012-14-26	294	610	518.23	1.18	…	…
Fx012-14-26	304	674	531.70	1.27	−18 (−49 to 7)	.183
M0–M32						
R012-20	298	971	625.11	1.55	…	…
Fx012-14-26	304	790	656.64	1.20	13 (−10 to 31)	.247
R012-14-26	294	728	635.09	1.15	…	…
Fx012-14-26	304	790	656.64	1.20	−16 (−47 to 9)	.226

Abbreviations: IVE, incremental vaccine efficacy; M, month; PY, person-years; CI, confidence interval.

**Figure 2.**
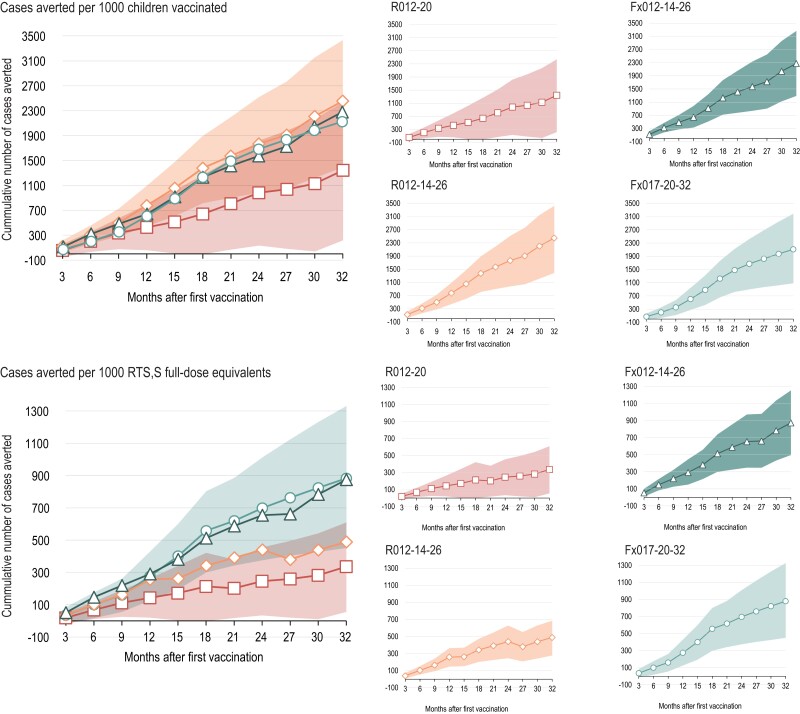
Vaccine impact expressed as cumulative number of clinical malaria cases averted (all episodes, secondary case definition) per 1000 children vaccinated or per 1000 RTS,S/AS01_E_ (RTS,S) full-dose equivalents administered, overall (exposed set). The estimated number of malaria cases averted was calculated as the area under the incidence curve of clinical malaria meeting the secondary case definitions captured in 3-month increments for each group (sum of the differences in incidence between the RTS,S and control groups summed over all the 3-month periods included in the period considered). Panels on the left represent the point estimates for all RTS,S groups at the 3-month interval, with the colored areas representing the 95% CIs with the highest upper limit and lowest lower limit, respectively, among the 4 estimates. For clarity, panels on the right represent values for each RTS,S group separately; areas represent 95% CIs. CI, confidence interval.

**Figure 3.**
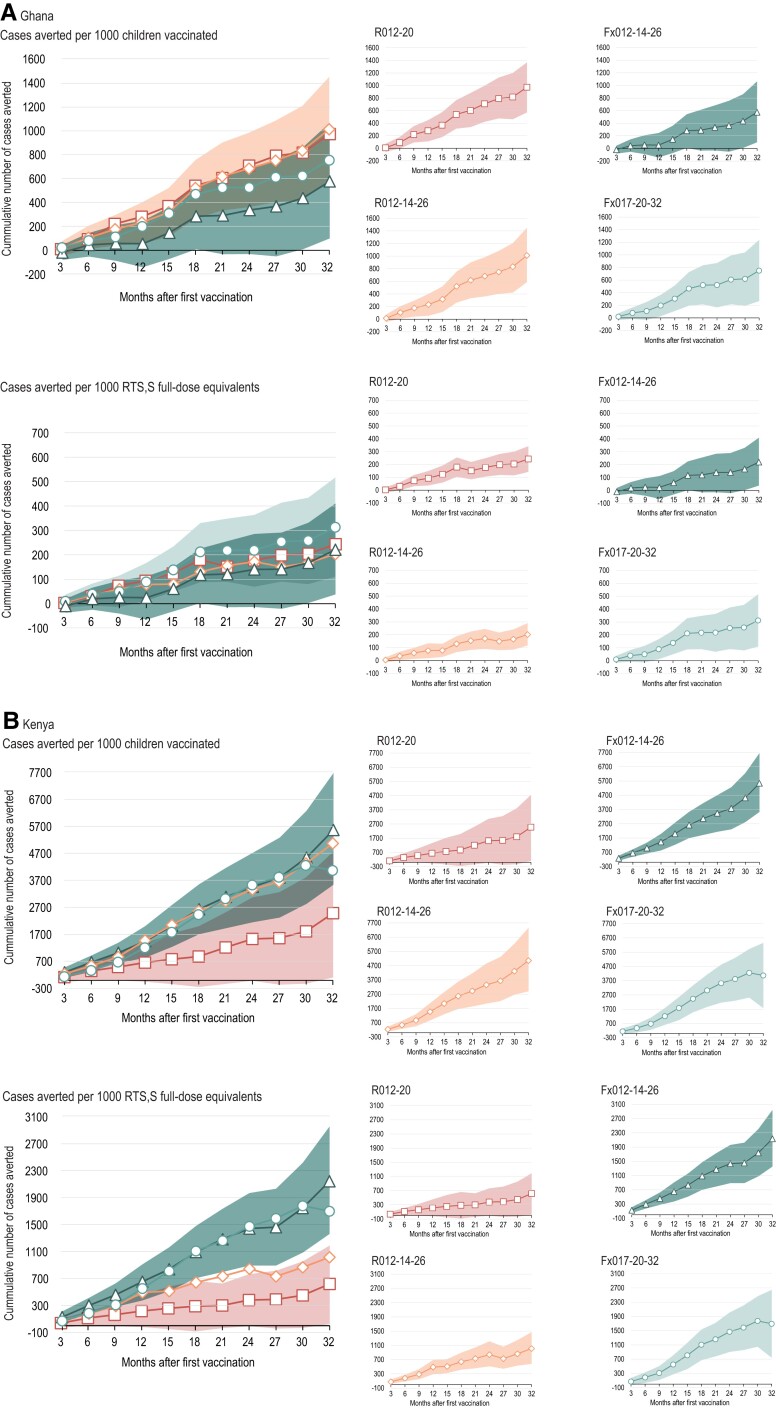
Vaccine impact expressed as cumulative number of clinical malaria cases averted (all episodes, secondary case definition) per 1000 children vaccinated or per 1000 RTS,S/AS01_E_ (RTS,S) full-dose equivalents administered in Ghana (*A*) and Kenya (*B*) (exposed set). The estimated number of malaria cases averted was calculated as the area under the incidence curve of clinical malaria meeting the secondary case definitions captured in 3-month increments for each group (sum of the differences in incidence between the RTS,S and control groups summed over all the 3-month periods included in the period considered). Panels on the left represent the point estimates for all RTS,S groups at the 3-month interval, with the colored areas representing the 95% CIs with the highest upper limit and lowest lower limit, respectively, among the 4 estimates. For clarity, panels on the right represent values for each RTS,S group separately; areas represent 95% CIs. CI, confidence interval.

In terms of cases of clinical malaria (all episodes meeting the secondary case definition) averted per 1000 children vaccinated as compared with the control group, overall vaccine impact over 32 months of follow-up was 1344 cases (R012-20 group), 2450 (R012-14-26 group), 2273 (Fx012-14-26 group), and 2112 (Fx017-20-32 group; Figure 2). The 95% CIs for the cumulative number of cases averted by 3-month periods overlapped among RTS,S groups at all time points (Supplementary Table 6). Over 32 months of follow-up, the numbers of malaria cases averted per 1000 RTS,S full-dose equivalents administered were 336 (R012-20 group), 490 (R012-14-26 group), 874 (Fx012-14-26 group), and 880 (Fx017-20-32 group). There was a trend for a higher number of malaria cases averted per 1000 RTS,S full-dose equivalents administered in the fractional-dose groups as compared with the full-dose RTS,S groups; however, 95% CIs overlapped. This trend was more pronounced in Kenya (Figure 3). The same trends, at a lower magnitude, were observed for the overall number of cases averted per 1000 full-dose equivalents when we consider that 4, 3, or 2 fractional doses of 0.1 mL are withdrawn from a 0.5-mL full-dose vaccine (Supplementary Figure 3, Supplementary Table 7).

Over 32 months of follow-up, all RTS,S regimens continued to be immunogenic. Anti-CS antibody responses peaked after the primary schedule and followed a normal antibody decay until boosting. Anti-CS antibody geometric mean concentrations increased following booster vaccinations in all groups but did not reach postprimary levels. Anti–hepatitis B surface antigen antibody responses followed a normal antibody decay pattern until boosting and increased after each RTS,S administration at higher levels than postprimary (Supplementary Figure 4, Supplementary Table 8). Overall, the mean anti-CS IgG antibody avidity index was similar across RTS,S groups and increased after each dose given, including fractional doses, consistent with results at M20 [11, Supplementary Table 8].

Solicited AEs after doses 3 and 4, unsolicited AEs within 30 days from any vaccination, SAEs, and AEs of special interest occurring up to M21 were previously reported [11]. The incidence of solicited AEs after dose 5 in the reactogenicity subset was low and comparable among groups R012-14-26, Fx012-14-26, and Fx017-20-32 (for which dose 5 was received in the M21–M33 period). From study start to M33, unsolicited AEs occurring within 30 days from any vaccination were documented after 40.2% to 40.6% of RTS,S doses and 50.5% of control vaccine doses. SAEs occurring within 30 days of each dose were reported after 0.8% to 1.3% of doses in RTS,S groups and 1.5% in the control group. Up to M33, 8 deaths were reported (Table 2); none were considered related to vaccination. The percentage of children with AEs of special interest continued to remain low and similar among all groups. Meningitis was identified for 1 child in each of the R012-20, R012-14-26, and Fx012-14-26 groups and for 2 children in each of the Fx017-20-32 and control groups. Severe malaria occurred in 18 to 27 children in the RTS,S groups and 44 children in the control group. Only 1 case of cerebral malaria (control group) was reported.

**Table 2.** Adverse Events up to Study Month 33

	R012-20		R012-14-26		Fx012-14-26		Fx017-20-32		Control	
	No.	% (95% CI)	No.	% (95% CI)	No.	% (95% CI)	No.	% (95% CI)	No.	% (95% CI)
**Solicited AEs over the 4-d follow-up period post–dose 5: reactogenicity subset** ^a^										
No. of doses received	…	…	42	…	39	…	42	…	…	…
Solicited local AEs										
Erythema			0	0 (0–8)	0	0 (0–9)	0	0 (0–8)		
Pain			4	10 (3–23)	1	3 (0–13)	0	0 (0–8)		
Swelling			0	0 (0–8)	1	3 (0–13)	1	2 (0–13)		
Solicited general AEs										
Drowsiness			0	0 (0–8)	1	3 (0–13)	0	0 (0–8)		
Irritability/fussiness			0	0 (0–8)	0	0 (0–9)	0	0 (0–8)		
Loss of appetite			0	0 (0–8)	1	3 (0–13)	0	0 (0–8)		
Fever			7	17 (7–31)	2	5 (1–17)	3	7 (1–19)		
Grade 3			0	0 (0–8)	1	3 (0–13)	0	0 (0–8)		
**Unsolicited AEs and SAEs within 30 d after any vaccination: exposed set**										
No. of doses received	1108	…	1364	…	1401	…	1421	…	855	…
Any unsolicited AE	446	40.3 (37.3–43.2)	550	40.3 (37.7–43.0)	569	40.6 (38.0–43.2)	571	40.2 (37.6–42.8)	432	50.5 (47.1–53.9)
Grade 3	11	1.0 (.5–1.8)	7	0.5 (.2–1.1)	10	0.7 (.3–1.3)	15	1.1 (.6–1.7)	8	0.9 (.4–1.8)
Related	18	1.6 (1.0–2.6)	35	2.6 (1.8–3.6)	10	0.7 (.3–1.3)	18	1.3 (.8–2.0)	6	0.7 (.3–1.5)
SAEs	13	1.2 (.6–2.0)	11	0.8 (.4–1.4)	14	1.0 (.5–1.7)	18	1.3 (.8–2.0)	13	1.5 (.8–2.6)
Related SAEs	3	0.3 (.1–.8)	0	0.0 (.0–.3)	0	0.0 (.0–.3)	2	0.1 (.0–.5)	0	0.0 (.0–.4)
**AEs of special interest and fatalities: exposed set)**										
No. of children	298	…	294	…	304	…	311	…	293	…
AEs of special interest										
Meningitis	1	0.3 (.0–1.9)	1	0.3 (.0–1.9)	1	0.3 (.0–1.8)	2	0.6 (.1–2.3)	2	0.7 (.1–2.4)
Seizure ^b^	5	1.7 (.5–3.9)	1	0.3 (.0–1.9)	0	0.0 (.0–1.2)	4	1.3 (.4–3.3)	4	1.4 (.4–3.5)
pIMD	0	0.0 (.0–1.2)	2	0.7 (.1–2.4)	0	0.0 (.0–1.2)	0	0.0 (.0–1.2)	0	0.0 (.0–1.3)
Severe malaria	18	6.0 (3.6–9.4)	19	6.5 (3.9–9.9)	21	6.9 (4.3–10.4)	27	8.7 (5.8–12.4)	44	15.0 (11.1–19.6)
Cerebral malaria	0	0.0 (.0–1.2)	0	0.0 (.0–1.2)	0	0.0 (.0–1.2)	0	0.0 (.0–1.2)	1	0.3 (.0–1.9)
Deaths ^c^	2	0.7 (.1–2.4)	1	0.3 (.0–1.9)	0	0.0 (.0–1.2)	4	1.3 (.4–3.3)	1	0.3 (.0–1.9)

Number and percentage indicate number of doses followed by at least 1 AE or number of children with at least 1 AE of special interest or fatality. Where not listed, no grade 3 AEs were reported in any group.

Abbreviations: AE, adverse event; CI, confidence interval; pIMD, potential immune-mediated disease; SAE, serious adverse event.

^a^The analysis included all children within the reactogenicity subset (ie, the first 25 children per country randomized to each group) who had safety data. All AEs in children not in the reactogenicity subset were reported as unsolicited AEs; all solicited local AEs were considered related to vaccination. Grade 3 solicited AEs were defined as erythema/swelling >20 mm, crying when limb is moved (pain), not eating at all (loss of appetite), preventing normal everyday activities (drowsiness, irritability/fussiness), temperature >39.0 °C (fever). The case definition of severe *P falciparum* malaria was the World Health Organization’s case definition of severe falciparum malaria for epidemiologic purposes (World Health Organization, http://apps.who.int/iris/handle/10665/162441).

^b^Within 30 days postvaccination.

^c^Fatal AEs were as follows: malaria (nonsevere) in an 11-month-old girl and a case of drowning in a 16-month-old boy (R012-20 group), a case of gastroenteritis in a 15-month-old girl (R012-14-26 group), a case of gastroenteritis in an 11-month-old girl, thermal burn in a 14-month-old girl, abdominal pain in a 14-month-old boy, a wall falling on a 6-month-old boy (Fx017-20-32 group), and severe malaria in a 7-month-old boy (control group).

## DISCUSSION

This ongoing study generates data on the protective efficacy against *P falciparum* malaria and vaccine impact of different RTS,S fractional- and full-dose regimens in field settings in 2 locations, Ghana and Kenya, with distinctly different malaria endemicity.

The study continues to demonstrate substantial VE for all 4 RTS,S regimens as compared with the control group over a longer follow-up (up to M32) and after at least 4 vaccine doses have been administered in all RTS,S groups. This is consistent with observations at M20 [11] and with the VE against clinical malaria estimated for the standard regimen in the phase 3 trial: 55.1% [15] and 37.4% [16] over 1 year of follow-up from doses 3 and 4, respectively. Long-term follow-up after the phase 3 trial indicated that a 4-dose regimen remained efficacious against malaria and severe malaria up to 7 years after administration of the first RTS,S dose [2, 17]. Although the current study was not able to demonstrate improved efficacy of fractional- vs full-dose regimens, no statistically significant differences were observed between regimens in terms of VE up to M20 [11] and now up to M32, indicating that the dosage and schedules of RTS,S may be flexible.

Vaccine impact (malaria cases averted/1000 children vaccinated) was comparable among all groups receiving full and fractional RTS,S doses over 20 months of follow-up (12 months after dose 3) [11]. The impact of vaccination is estimated in clinical trials by calculating the difference in the number of malaria cases between vaccine groups and the control group and is usually expressed as the number of malaria cases averted per 1000 children vaccinated. Although the number of malaria cases that can be prevented by a malaria intervention is highly dependent on the background malaria incidence in the population, this measure of impact per 1000 children vaccinated allows comparison among vaccines evaluated in clinical trials. However, in a study involving multiple study arms where each arm receives a different number of vaccine doses that may be full or fractional, the total volume of vaccine used to generate the impact in each group should be considered. Over the 32 months of follow-up reported here, children in the R012-20 and R012-14-26 groups received 4 and 5 full-dose RTS,S vaccinations, respectively, whereas children in the Fx012-14-26 group received 2 full and 3 fractional RTS,S doses (or 2.6 full-dose equivalents). Similarly, children in the Fx017-20-32 group received 2 full and 2 fractional RTS,S doses (or 2.4 full-dose equivalents). When we account for the volume of vaccine administered, the vaccine impact of fractional-dose regimens tended to be more substantial as compared with the full-dose regimens, regardless of the number of fractional doses withdrawn (n = 2 to 5).

This difference tended to increase over time and to be more pronounced in Kenya. Nevertheless, extracting 5 fractional doses from a 0.5-mL full-dose vaccine volume may represent a best-case scenario that may not be fully reproducible in real-life settings. RTS,S is available as 2-dose vials, so vaccine wastage with fractional dosing may need to be considered. Real-world data available for the use of fractional doses of poliovirus vaccine show that in routine immunization settings in India, a median of 48 of a maximum 50 intradermal fractional doses could be extracted from 10 full-dose vials [18]. In Pakistan, wastage rates of 6% to 10% (depending on the intradermal 0.5-mL device used) were estimated for the use of 5 full-dose vials [19]. Wastage might be minimal for RTS,S. The use of fractional RTS,S doses could allow for more children to be vaccinated with the same amount of vaccine, potentially improving the cost-effectiveness of the program. However, implementing a hybrid vaccination regimen (with full and fractional doses) can present more operational challenges than when only fractional doses are administered, as shown from previous experiences with the poliovirus vaccine [20], so the latter approach should also be evaluated. Although our study was not designed to assess hybrid or fractional-dose regimens as a dose-sparing strategy, these initial results indicate that administration of fractional doses could be considered during discussion on the rollout of RTS,S to maximize the public health impact that can be generated with the available vaccine. This could reduce the overall cost of a vaccination program that will be predominantly implemented in low-income countries and is reliant on public funding.

The use of a fractional instead of a full dose as a dose-sparing method to mitigate the risk of limited vaccine supply or stockouts has been successfully applied and authorized by the WHO and other regulatory bodies (eg, smallpox vaccine used against mpox [21], yellow fever [5]) and supported by modeling [22].

Other strategies for malaria prevention currently under evaluation by the WHO [23] may become more viable with the use of fractional doses. For instance, fractional doses could be considered as booster and subsequent annual doses after full-dose primary vaccinations or for seasonal vaccination with RTS,S in combination with SMC. However, the current study evaluates the use of fractional dosing only for the third and subsequent doses, and data up to M50 are still needed to confirm the long-term efficacy and immune responses elicited by the fractional-dose regimens. Additionally, the use of vaccination regimen with 2 different dosages may not only complicate vaccine administration logistics but also increase the risk for administration errors, although our study indicates no safety concerns in case of the erroneous administration of a full instead of fractional dose. More studies may be needed to assess whether fractional doses given throughout an RTS,S regimen can provide equivalent benefit against malaria.

Immune responses induced by the fractional-dose regimens continue to be comparable to those in full-dose groups, as observed at M21 [11]. A trend for diminished peak anti-CS antibody levels was observed after subsequent booster doses in the full- and fractional-dose groups, but the 95% CIs for anti-CS antibody geometric mean concentrations after doses 4 and 5 overlapped among groups, and prebooster anti-CS geometric mean concentrations were similar.

This lower magnitude of peak anti-CS antibody responses following booster and subsequent RTS,S full doses was previously observed [4, 16, 24]; however, it did not appear to translate into a substantial decrease in VE in seasonal settings [4, 24]. Anti-CS antibody avidity continued to be similar between the fractional- and full-dose groups at M33 as seen at M20 [11], and we observed an increase in avidity following each administered RTS,S dose, decreasing in magnitude over time after every dose. In a previous controlled human malaria infection study in malaria-naive adults, an Fx017 regimen led to increased anti-CS antibody avidity when compared with an R012 regimen [10, 25], although in a second controlled human malaria infection study, no difference was observed between protected and unprotected individuals [9]. In line with our observations, a phase 2 pediatric efficacy trial evaluating full-dose R012 and Fx017 RTS,S regimens showed that avidity of anti-CS antibodies after the third dose was similar between these regimens [26]. Other pediatric RTS,S studies showed that anti-CS protein IgG concentrations and avidity contribute to protection against clinical malaria but are not predictors of VE [26, 27].

All RTS,S regimens continued to be well tolerated, with a similar incidence of solicited and unsolicited AEs to that observed in the phase 3 trial in African children aged 5 to 17 months [15]. No trend for increased reactogenicity was observed with subsequent booster doses in RTS,S groups. The frequency of SAEs remained low even after 5 RTS,S doses, consistent with previous observations [4]. A substantially lower incidence of severe malaria cases was observed in all RTS,S groups (6.0%–8.7%) as compared with the control group (15.0%), similar to the significant reduction in severe malaria previously reported after 4 RTS,S full doses [16, 28]. Although not assessed in our study, given the small sample size, this suggests that fractional-dose regimens could provide benefit against severe malaria as well. There continues to be no indication of an increased risk for meningitis or cerebral malaria in the RTS,S groups vs the control group. This is similar to observations from the ongoing WHO-coordinated pilot program in areas of Ghana, Kenya, and Malawi [29], following the routine vaccination of >1 million children.

In addition to previously discussed study limitations [11], we note that the analysis of the number of cases averted per 1000 RTS,S full-dose equivalents was performed on the exposed set, which included children receiving at least 1 dose. However, the relatively high level of compliance to vaccination suggests a low effect on the vaccine impact results. All results should be interpreted with caution, as the primary study objective (superior efficacy of Fx012 vs R012) was not met at M21 and the secondary objectives are to be assessed in a hierarchical manner [11].

In conclusion, all RTS,S regimens continued to provide similar substantial protection against clinical malaria over 32 months after the first dose. Expressing malaria cases averted per 1000 vaccine doses administered may be a relevant measure to complement vaccine impact evaluations when different vaccine regimens and doses are administered across study arms. Although superiority of fractional- vs full-dose regimens was not demonstrated and the complementary impact analyses were post hoc, we showed an overall trend of higher vaccine impact for fractional- vs full-dose regimens of RTS,S, supporting flexibility around timing and dosing in the vaccination schedule. This vaccine impact analysis provides important and timely information on the potential value of fractional dosing for future implementation considerations. The forthcoming final analysis at M50 will further inform the long-term effects of fractional RTS,S dosing.

## Supplementary Data

Supplementary materials are available at *The Journal of Infectious Diseases* online (http://jid.oxfordjournals.org/). Supplementary materials consist of data provided by the author that are published to benefit the reader. The posted materials are not copyedited. The contents of all supplementary data are the sole responsibility of the authors. Questions or messages regarding errors should be addressed to the author.

## Supplementary Material

jiae075_Supplementary_Data

## Notes

***Acknowledgments***. The authors and trial partners thank the study participants and staff for their participation and support of malaria clinical research. The authors thank the following for their contribution to the study: Karen Ivinson (PATH's Center for Vaccine Innovation and Access, Geneva, Switzerland); Cristina Cravcenco and Erik Jongert (GSK, Wavre, Belgium); David Sambian, Albert Agordo Dornudo, Lydia Nana Badu, Kwame Akoi, Evans Antwi, and Nii Ayi Tagoe (Kwame Nkrumah University of Science and Technology/Agogo Presbyterian Hospital, Agogo, Ghana); and Kelvin Onoka, Kevin K’Orimba, and Lilian Otieno (Centre for Global Health Research, Kenya Medical Research Institute, Kisumu, Kenya). The authors are also grateful for the help and dedication of the following groups and individuals: Obed Affum Addo, Nelson Batumah, Collins Paa Yeboah, Reindolf Oteng, and Samuel Boateng (Ghana); Kennedy Odhiambo Oruenjo (Siaya county director of public health), the Siaya County health management team, the Siaya County Referral Hospital medical superintendent (Kenya); HJFMRI Kisumu (Kenya) for administrative support provided during the study and for partnership with KEMRI; Som Shankar Guin, the GSK global and local operations teams, and Hildegarde Lemaire (GSK); Anna Jameh, who worked as part of the study team during her internship at GSK; the PATH team, including Laina Mercer, Scott Gregory, Sahlah Dubel; and the team at KEMRI-WRAIR Malaria Diagnostic Center. We thank the US Centers for Disease Control and Prevention for its in-kind salary support for A. M. S. and N. W. and KEMRI for its in-kind salary support for S. K. K. The authors thank Akkodis Belgium in care of GSK for medical writing and design support (Petronela M. Petrar) and manuscript coordination.

***Authors contributions*.** All authors participated in the design, implementation, or analysis of the study. All authors participated in the interpretation of study data and the development of this article. All authors had full access to the analyses and data and granted their final approval of the manuscript before submission. A. Bo. and O. O.-A. accessed and verified all data used in the study. All authors had final responsibility for the decision to submit for publication.

***Data sharing*.** Anonymized individual participant data and study documents will be available at study end, upon request for further research from www.clinicalstudydatarequest.com (study ID: 204889).

***Financial support*.** Funding for this trial was provided by GlaxoSmithKline Biologicals SA (study sponsor); PATH, an international public health organization, through grants awarded to PATH's Malaria Vaccine Initiative by the Bill and Melinda Gates Foundation (grant INV-007217) and the German Federal Ministry of Education and Research (Reference No.: 2020 62 156, administered through the KfW Development Bank). Experimentalproducts were provided by GlaxoSmithKline Biologicals SA.
