# Supplementary material for: Could Less Be More? Accounting for Fractional-Dose Regimens and Different Number of Vaccine Doses When Measuring the Impact of the RTS,S/AS01E Malaria Vaccine
Source: J Infect Dis. 2024 Mar 4;230(2):e486–95. doi: 10.1093/infdis/jiae075 (PMC11326831; doi:10.1093/infdis/jiae075)
Supplement: jiae075_Supplementary_Data [file jiae075_supplementary_data.docx]

# Supplementary material

# Supplementary Figure 1. Study design overview


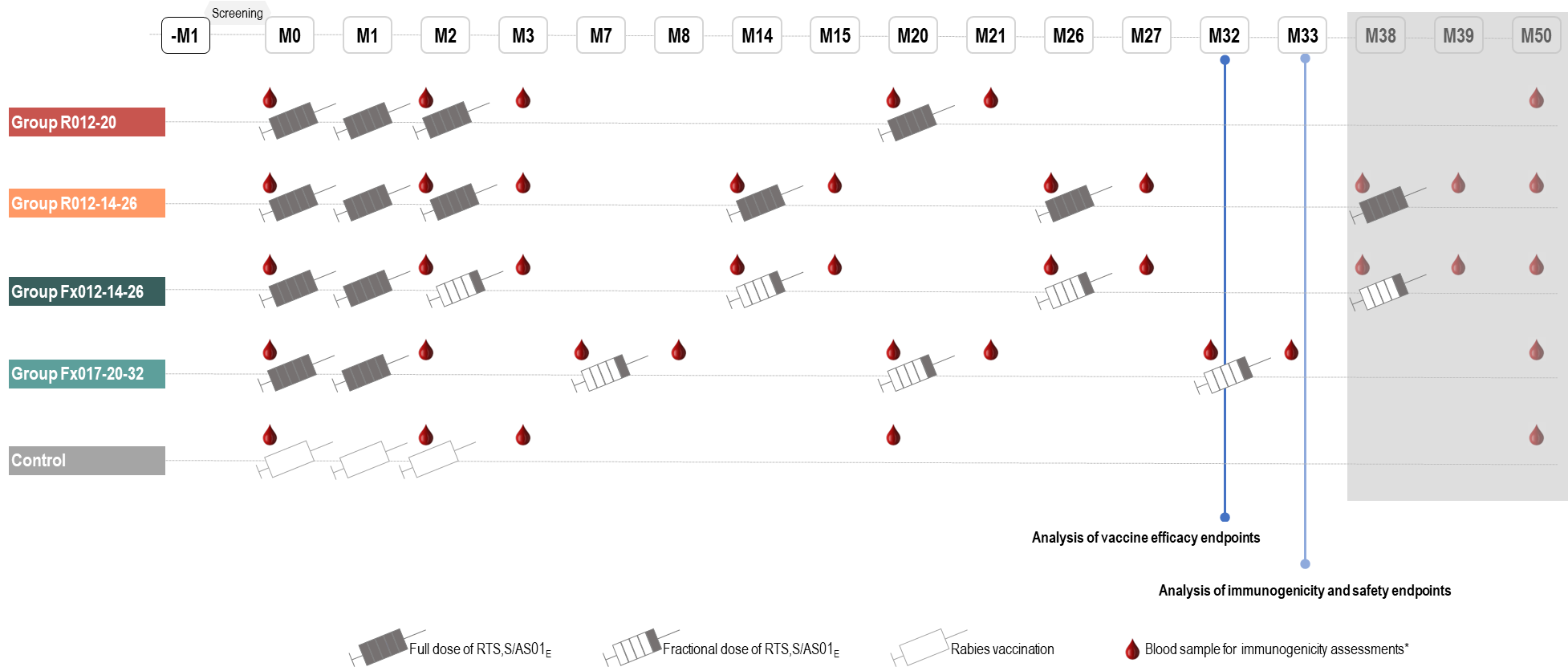


M, month.

Notes: *Additional blood samples were collected in the reactogenicity/immunogenicity subset (first 50 children [25 per country] randomised into each group), for the evaluation of biochemistry and haematology parameters (pre-dose 3, seven days post-dose 3 and 30 days post-dose 3).

Blue and light blue lines indicate the timepoints at which analyses reported in the current manuscript were carried out. The greyed-out area indicates the study period for which results are not yet available.

**Supplementary Figure 2. Flowchart for study participants up to study month 33**


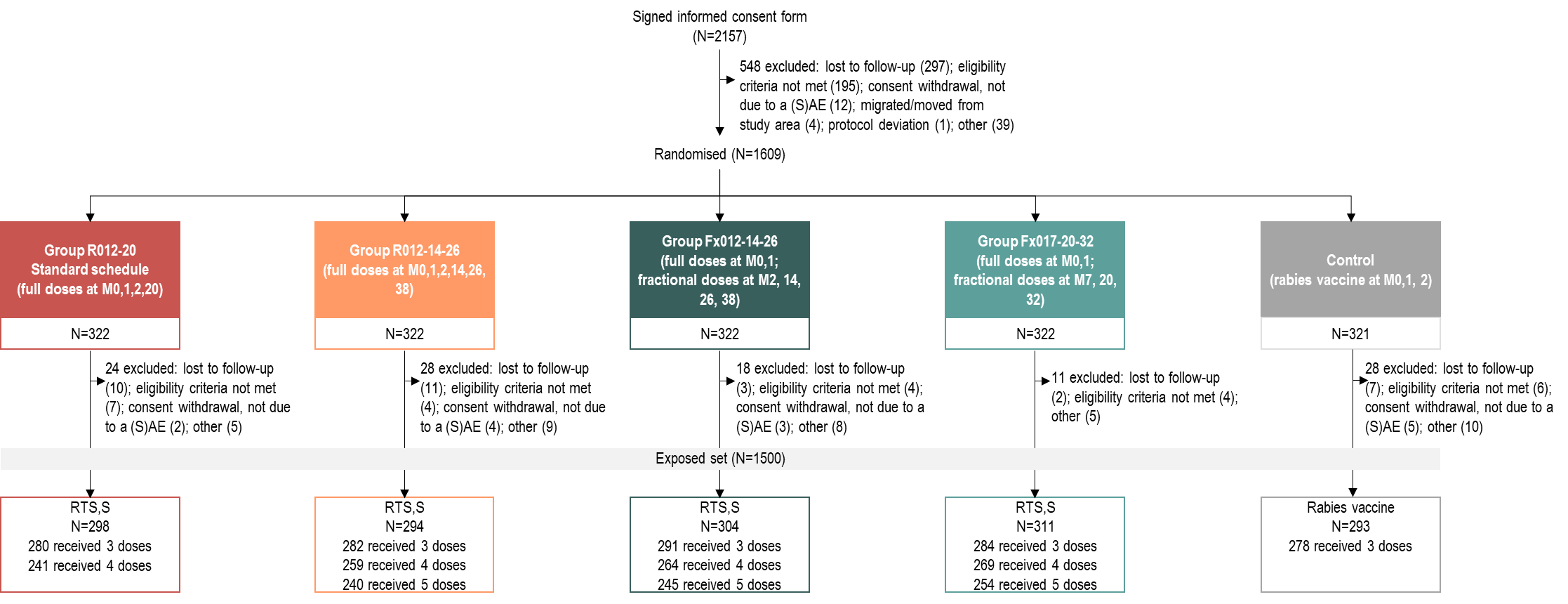


N, number of children; M, month; (S)AE, (serious) adverse event.

Note: Other reasons for exclusion included: not attending first visit as scheduled (within 28 days from screening), incomplete screening procedures, one parent declining participation, weight-for-height Z-score less than -2 standard deviations of the international standard, low haemoglobin concentration, and recruitment target reached.

# Supplementary Figure 3. Vaccine impact expressed as cumulative number of clinical malaria cases averted (all episodes, secondary case definition) per 1000 RTS,S full-dose equivalents administered when considering that only 4, 3, or 2 fractional doses (0.1 mL) can be withdrawn from a 0.5 mL full-dose vaccine, overall (exposed set)


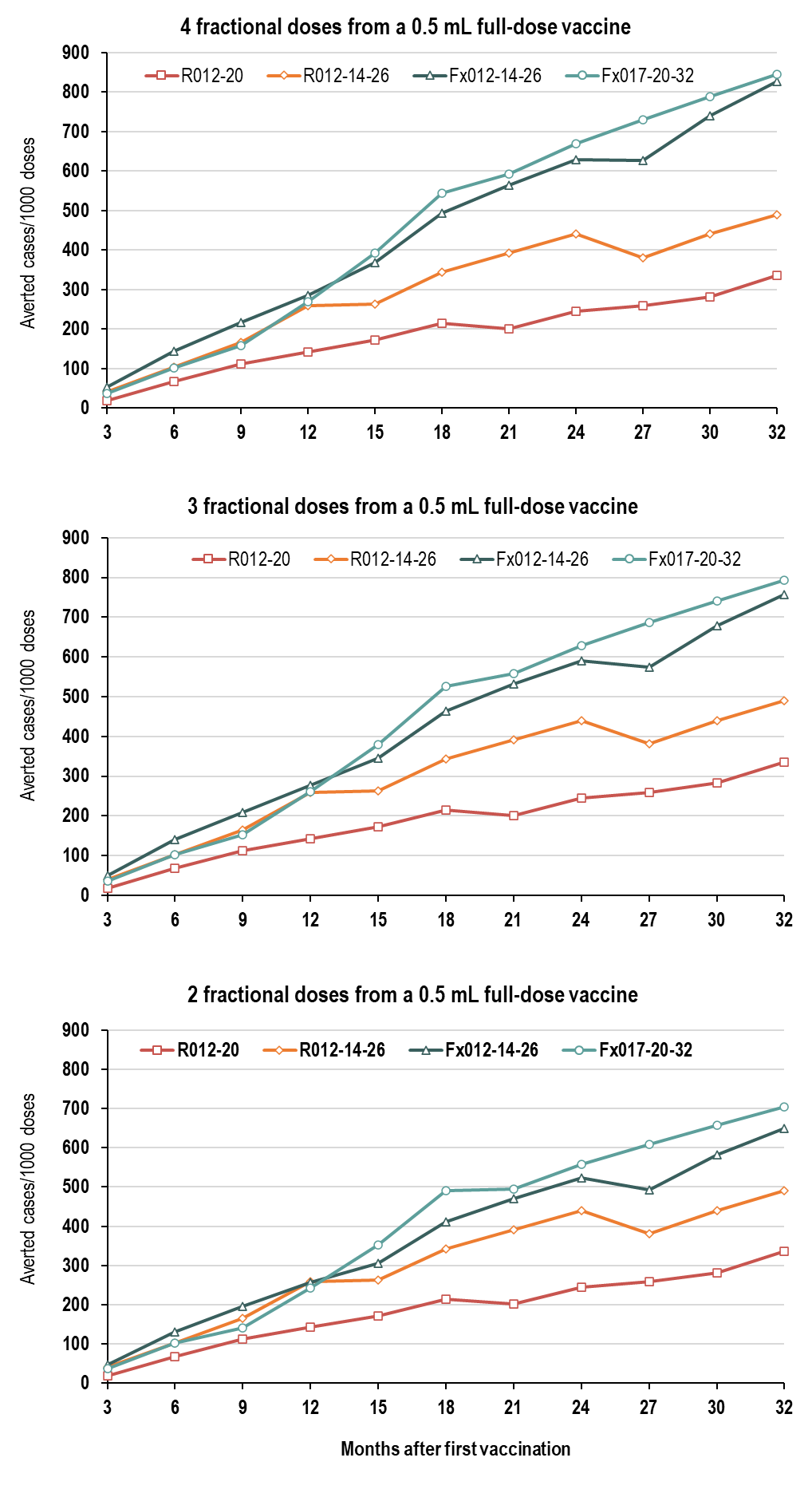


Note: 95% Confidence intervals are provided in Supplementary Table 7.

# Supplementary Figure 4. Immune responses to vaccination: anti-CS antibody GMCs (a, a’) and anti-HBs antibody GMCs (b, b’), by timepoint and by month from first dose (immunogenicity subset, per-protocol set for immunogenicity)


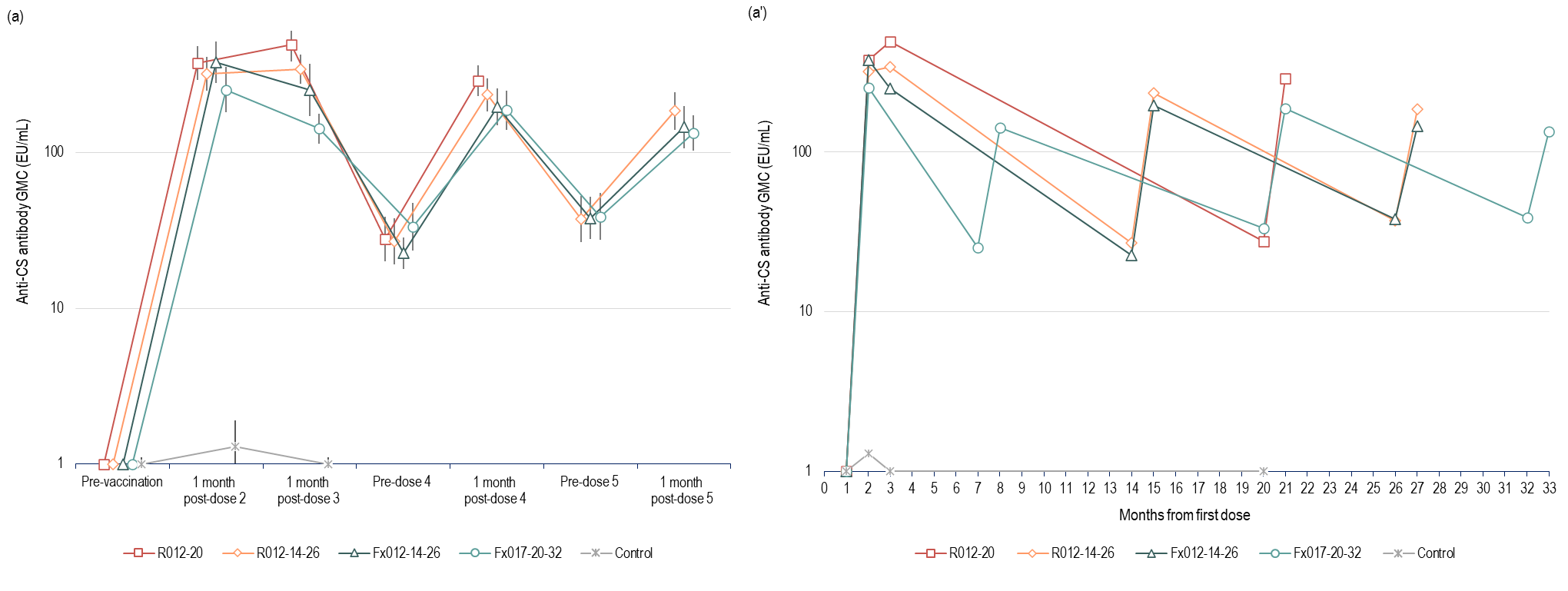


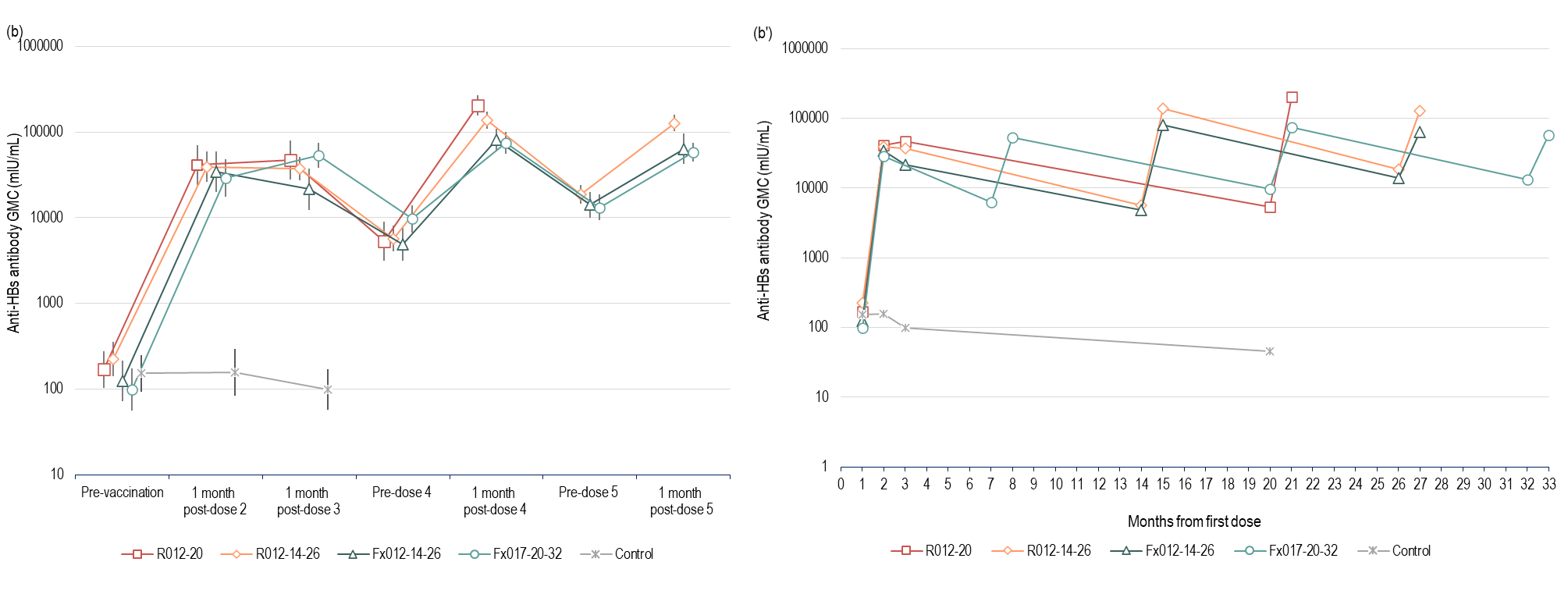


CS, circumsporozoite protein; GMC, geometric mean concentration; EU, enzyme-linked immunosorbent assay units; HBs, anti-hepatitis B surface antigen; IU, international units.

Note: Error bars represent 95% confidence intervals.

# Supplementary Table 1. Full list of study objectives

| **Objective** | **Note** |
| --- | --- |
| **Primary objective:** |  |
| To demonstrate the superiority of a 3-dose schedule of RTS,S/AS01_E_ with a fractional third dose at month 2 compared to a standard schedule of RTS,S/AS01_E_ with three full doses in terms of vaccine efficacy against clinical malaria (primary case definition) over 12 months post-dose 3. | Reported in [1]. |
| **Secondary objectives:** |  |
| **Efficacy:**  To assess the incremental vaccine efficacy against clinical malaria of a schedule with a fractional third dose at month 2 versus a schedule with three full doses (primary and secondary case definitions*) over 12 months post-dose 3.  To assess the incremental vaccine efficacy against clinical malaria, over 7, and 12 months post-dose 3, of a schedule with a fractional third dose at month 7 versus a schedule with a fractional third dose at month 2.  To assess the incremental vaccine efficacy against clinical malaria, over 7, and 12 months post-dose 3, of a schedule with a fractional third dose at month 7 versus a schedule with three full doses.  To assess the incremental vaccine efficacy against clinical malaria, over 12 months post-dose 4, post-dose 5 and post-dose 6 of a schedule with a fractional third dose at month 2 and yearly fractional doses versus a schedule with full doses at month 0, month 1, month 2, and yearly full doses.  To assess the incremental vaccine efficacy against clinical malaria, over 12 months post-dose 4, of a schedule with a fractional third dose at month 7 versus a standard schedule with four full doses at 0,1,2,20 months.  To assess the vaccine efficacy and impact of each RTS,S/AS01_E_ schedule over the entire study period by measuring the efficacy against clinical malaria at months 14, 20, 26, 32, 38, and 50.  To assess the prevalence of *P. falciparum* infections of each RTS,S/AS01_E_ schedule at cross-sectional visits (monthly from month 0-20 and every three months thereafter until study end).  To assess the vaccine efficacy against incident *P. falciparum* infections defined by positive blood slide over the entire study period. | Reported in [1].  Reported in [1].  Reported in [1].  Data at 12 months post-dose 4 is presented here. Other data will be reported elsewhere.  Data up to 12 months post-dose 4 is reported here. Other data will be reported elsewhere.  Data at months 26 and 32 are reported here. Other data will be reported elsewhere.  Partially reported in [1]. Other data to be reported elsewhere.  Partially reported in [1]. Other data to be reported elsewhere. |
| **Immunogenicity:**  To describe the antibody response to the anti-circumsporozoite protein of *P. falciparum* (anti-CS) for each schedule.  To describe the antibody response to the hepatitis B surface antigen (anti-HBs) for each schedule. | Data up to month 33 (at 1 month post-dose 5) are reported in this paper. |
| **Safety:**  To assess the safety of RTS,S/AS01_E_ for each schedule in terms of serious adverse events (SAEs), unsolicited adverse events (AEs) and AEs of specific interest.  To assess the reactogenicity of RTS,S/AS01_E_ in terms of solicited local and general AEs.  To assess the safety of RTS,S/AS01_E_ in terms of biochemistry (alanine aminotransferase, creatinine) and haematology (haemoglobin, white blood cells, platelets) parameters. | Data up to month 20 are reported in [1] and up to month 33 in this paper. |
| **Tertiary objective** |  |
| To describe the anti-CS antibody response in terms of avidity. | Data up to month 20 are reported in [1] and up to month 33 in this paper. |

Reference:

1. Samuels AM, Ansong D, Kariuki SK, et al. Efficacy of RTS,S/AS01_E_ malaria vaccine administered according to different full, fractional, and delayed third or early fourth dose regimens in children aged 5-17 months in Ghana and Kenya: an open-label, phase 2b, randomised controlled trial. Lancet Infect Dis 2022; 22:1329-42.

Note: *The primary case definition was *P. falciparum* asexual parasitaemia >5000 parasites/μL AND presence of fever (axillary temperature ≥37.5°C) at the time of presentation AND occurring in a child who is brought for treatment to a healthcare facility. The secondary case definition was *P. falciparum* asexual parasitaemia >0 parasites/μL AND presence of fever (axillary temperature ≥37.5°C) at the time of presentation or history of fever within 24 hours of presentation AND occurring in a child who is brought for treatment to a healthcare facility.

**Supplementary Table 2. Baseline characteristics of study participants, overall and by country (exposed set)**

|  | **Group R012-20** | **Group R012-14-26** | **Group Fx012-14-26** | **Group Fx017-20-32** | **Control group** |
| --- | --- | --- | --- | --- | --- |
| N | 298 | 294 | 304 | 311 | 293 |
| Ghana | 153 | 151 | 148 | 151 | 147 |
| Kenya | 145 | 143 | 156 | 160 | 146 |
| Age at first vaccination, months (mean±SD) | 10.2±3.9 | 10.3±3.8 | 10.5±4.0 | 10.2±3.8 | 10.5±3.9 |
| Ghana | 9.7±3.9 | 10.4±4.0 | 10.1±4.0 | 10.2±4.1 | 10.4±4.0 |
| Kenya | 10.7±3.7 | 10.1±3.6 | 10.9±4.0 | 10.2±3.6 | 10.7±3.8 |
| Male, n (%) | 179 (60.1) | 140 (47.6) | 132 (43.4) | 148 (47.6) | 141 (48.1) |
| Ghana | 93 (60.8) | 68 (45.0) | 64 (43.2) | 76 (50.3) | 65 (44.2) |
| Kenya | 86 (59.3) | 72 (50.3) | 68 (43.6) | 72 (45.0) | 76 (52.1) |
| Length, cm (mean±SD) | 70.7±5.3 | 71.0±5.3 | 70.8±5.2 | 70.7±5.1 | 71.2±5.3 |
| Ghana | 70.4±5.6 | 71.4±5.3 | 71.0±5.6 | 71.4±5.6 | 71.8±5.4 |
| Kenya | 71.0±5.0 | 70.7±5.2 | 70.6±4.7 | 70.1±4.5 | 70.7±5.1 |
| Weight, kg (mean±SD) | 8.5±1.3 | 8.5±1.5 | 8.5±1.4 | 8.4±1.4 | 8.4±1.3 |
| Ghana | 8.2±1.4 | 8.3±1.4 | 8.3±1.4 | 8.3±1.6 | 8.3±1.4 |
| Kenya | 8.8±1.3 | 8.7±1.6 | 8.6±1.3 | 8.5±1.2 | 8.5±1.3 |
| Baseline haemoglobin, g/dL (mean±SD) | 10.1±1.1 | 10.3±1.1 | 10.4±1.1 | 10.3±1.1 | 10.3±1.1 |
| Ghana | 10.5±1.1 | 10.7±1.0 | 10.7±1.1 | 10.6±1.0 | 10.7±1.0 |
| Kenya | 9.7±1.1 | 9.9±1.1 | 10.1±1.1 | 10.0±1.0 | 9.9±1.2 |

N, number of children in each group; SD, standard deviation; n (%), number (percentage) of children in each category.

# Supplementary Table 3. Summary of vaccine efficacy against all clinical malaria episodes (primary or secondary case definitions) over different time periods up to M32, overall

| **Time period** | **Group** | **N** | **n** | **T year** | **n/T** | **VE %** | **95% CI** | **p-value** |
| --- | --- | --- | --- | --- | --- | --- | --- | --- |
|  |  |  |  |  |  |  |  |  |
| **VE against all clinical malaria episodes meeting the primary case definition, exposed set** | | | | | | | | |
| M0–M26 | Control | 293 | 630 | 518.97 | 1.21 |  |  |  |
|  | R012-20 | 298 | 429 | 521.15 | 0.82 | 43 | 28; 54 | <0.001 |
|  | R012-14-26 | 294 | 356 | 527.76 | 0.67 | 51 | 38; 61 | <0.001 |
|  | Fx012-14-26 | 304 | 414 | 541.60 | 0.76 | 41 | 26; 53 | <0.001 |
|  | Fx017-20-32 | 311 | 393 | 560.37 | 0.70 | 48 | 35; 59 | <0.001 |
| M0–M32 | Control | 293 | 777 | 639.46 | 1.22 |  |  |  |
|  | R012-20 | 298 | 541 | 641.39 | 0.84 | 42 | 27; 54 | <0.001 |
|  | R012-14-26 | 294 | 422 | 646.75 | 0.65 | 54 | 42; 64 | <0.001 |
|  | Fx012-14-26 | 304 | 482 | 668.28 | 0.72 | 45 | 31; 56 | <0.001 |
|  | Fx017-20-32 | 311 | 499 | 696.53 | 0.72 | 47 | 34; 58 | <0.001 |
| **VE against all clinical malaria episodes meeting the primary case definition, per-protocol set** | | | | | | | | |
| M14–M26 | Control | 243 | 334 | 223.63 | 1.49 |  |  |  |
|  | R012-14-26 | 245 | 190 | 218.90 | 0.87 | 51 | 35; 63 | <0.001 |
|  | Fx012-14-26 | 251 | 198 | 222.86 | 0.89 | 49 | 32; 62 | <0.001 |
| M20–M32 | Control | 231 | 269 | 215.48 | 1.25 |  |  |  |
|  | R012-20 | 230 | 185 | 213.88 | 0.86 | 43 | 21; 59 | <0.001 |
|  | Fx017-20-32 | 261 | 177 | 242.53 | 0.73 | 48 | 30; 61 | <0.001 |
| M7.5–M26 | Control | 236 | 448 | 321.30 | 1.39 |  |  |  |
|  | Fx017-20-32 | 274 | 288 | 377.56 | 0.76 | 55 | 42; 65 | <0.001 |
| M2.5–M26 | Control | 265 | 545 | 445.11 | 1.22 |  |  |  |
|  | R012-20 | 259 | 384 | 439.39 | 0.87 | 41 | 25; 54 | <0.001 |
|  | R012-14-26 | 264 | 323 | 450.32 | 0.72 | 50 | 36; 61 | <0.001 |
|  | Fx012-14-26 | 271 | 357 | 456.87 | 0.78 | 43 | 27; 55 | <0.001 |
| M7.5-M32 | Control | 236 | 580 | 429.52 | 1.35 |  |  |  |
|  | Fx017-20-32 | 274 | 386 | 506.30 | 0.76 | 54 | 41; 65 | <0.001 |
| M2.5–M32 | Control | 265 | 680 | 560.59 | 1.21 |  |  |  |
|  | R012-20 | 259 | 487 | 549.06 | 0.89 | 41 | 24; 53 | <0.001 |
|  | R012-14-26 | 264 | 383 | 562.80 | 0.68 | 54 | 41; 64 | <0.001 |
|  | Fx012-14-26 | 271 | 419 | 569.52 | 0.74 | 46 | 31; 58 | <0.001 |
| **VE against all clinical malaria episodes meeting the secondary case definition, per-protocol set** | | | | | | | |  |
| M14–M26 | Control | 243 | 543 | 215.72 | 2.52 |  |  |  |
|  | R012-14-26 | 245 | 329 | 213.77 | 1.54 | 50 | 36; 61 | <0.001 |
|  | Fx012-14-26 | 251 | 318 | 218.31 | 1.46 | 50 | 36; 61 | <0.001 |
| M20–M32 | Control | 231 | 429 | 209.48 | 2.05 |  |  |  |
|  | R012-20 | 230 | 334 | 208.35 | 1.60 | 39 | 19; 54 | <0.001 |
|  | Fx017-20-32 | 261 | 309 | 237.59 | 1.30 | 45 | 28; 58 | <0.001 |
| M7.5–M26 | Control | 236 | 732 | 310.53 | 2.36 |  |  |  |
|  | Fx017-20-32 | 274 | 501 | 369.47 | 1.36 | 54 | 42; 64 | <0.001 |
| M2.5–M26 | Control | 265 | 888 | 432.09 | 2.06 |  |  |  |
|  | R012-20 | 259 | 695 | 427.52 | 1.63 | 37 | 21; 49 | <0.001 |
|  | R012-14-26 | 264 | 550 | 441.83 | 1.24 | 50 | 38; 60 | <0.001 |
|  | Fx012-14-26 | 271 | 576 | 448.52 | 1.28 | 44 | 31; 55 | <0.001 |
| M7.5–M32 | Control | 236 | 933 | 416.15 | 2.24 |  |  |  |
|  | Fx017-20-32 | 274 | 667 | 495.66 | 1.35 | 52 | 40; 62 | <0.001 |
| M2.5–M32 | Control | 265 | 1097 | 544.76 | 2.01 |  |  |  |
|  | R012-20 | 259 | 876 | 534.32 | 1.64 | 37 | 21; 49 | <0.001 |
|  | R012-14-26 | 264 | 659 | 552.29 | 1.19 | 53 | 41; 63 | <0.001 |
|  | Fx012-14-26 | 271 | 683 | 559.56 | 1.22 | 47 | 34; 57 | <0.001 |

M, month; N, number of participants in each group contributing to the considered evaluation period; n, number of episodes included in each group; T (year), person years at risk; n/T, person year rate in each group; VE, vaccine efficacy; CI, confidence interval.

Notes: The per-protocol set for efficacy included children who received all three first vaccinations as per protocol and who contributed to efficacy surveillance starting 14 days post-dose 3. The per-protocol set comprised 259 children in the R012-20 group, 264 children in the R012-14-26 group, 271 children in the Fx012-14-26 group, 273 children in the Fx017-20-32 group, and 265 children in the control group.

Since the primary objective of the study (superiority of vaccine efficacy against clinical malaria [primary case definition] of the Fx012 versus R012 regimen at 12 months post-dose 3) was not demonstrated, any group comparisons should be interpreted with caution.

# Supplementary Table 4. Summary of vaccine efficacy against all clinical malaria episodes (primary or secondary case definitions) over different time periods up to M32, by country

| **Time period** | **Group** | **N** | **n** | **T year** | **n/T** | **VE %** | **95% CI** | **p-value** |
| --- | --- | --- | --- | --- | --- | --- | --- | --- |
|  |  |  |  |  |  |  |  |  |
| **VE against all clinical malaria episodes meeting the primary case definition, exposed set** | | | | | | | | |
| M0–M26 |  |  |  |  |  |  |  |  |
| Ghana | Control | 147 | 120 | 286.41 | 0.42 |  |  |  |
|  | R012-20 | 153 | 43 | 283.87 | 0.15 | 66 | 45; 78 | <0.001 |
|  | R012-14-26 | 151 | 50 | 283.47 | 0.18 | 61 | 38; 75 | <0.001 |
|  | Fx012-14-26 | 148 | 87 | 285.52 | 0.30 | 32 | -2; 54 | 0.064 |
|  | Fx017-20-32 | 151 | 66 | 292.45 | 0.23 | 52 | 25; 69 | 0.001 |
| Kenya | Control | 146 | 510 | 232.55 | 2.19 |  |  |  |
|  | R012-20 | 145 | 386 | 237.28 | 1.63 | 30 | 9; 46 | 0.007 |
|  | R012-14-26 | 143 | 306 | 244.30 | 1.25 | 46 | 30; 59 | <0.001 |
|  | Fx012-14-26 | 156 | 327 | 256.08 | 1.28 | 45 | 29; 58 | <0.001 |
|  | Fx017-20-32 | 160 | 327 | 267.92 | 1.22 | 47 | 31; 59 | <0.001 |
| M0–M32 |  |  |  |  |  |  |  |  |
| Ghana | Control | 147 | 149 | 359.54 | 0.41 |  |  |  |
|  | R012-20 | 153 | 58 | 353.10 | 0.16 | 63 | 43; 76 | <0.001 |
|  | R012-14-26 | 151 | 56 | 351.02 | 0.16 | 65 | 45; 78 | <0.001 |
|  | Fx012-14-26 | 148 | 97 | 354.87 | 0.27 | 39 | 10; 59 | 0.014 |
|  | Fx017-20-32 | 151 | 78 | 367.05 | 0.21 | 53 | 29; 69 | <0.001 |
| Kenya | Control | 146 | 628 | 279.92 | 2.24 |  |  |  |
|  | R012-20 | 145 | 483 | 288.29 | 1.68 | 29 | 7; 45 | 0.011 |
|  | R012-14-26 | 143 | 366 | 295.73 | 1.24 | 48 | 32; 61 | <0.001 |
|  | Fx012-14-26 | 156 | 385 | 313.42 | 1.23 | 48 | 32; 60 | <0.001 |
|  | Fx017-20-32 | 160 | 421 | 329.47 | 1.28 | 45 | 28; 58 | <0.001 |
| **VE against all clinical malaria episodes meeting the primary case definition, per-protocol set** | | | | | | | | |
| M14–M26 | |  |  |  |  |  |  |  |
| Ghana | Control | 136 | 67 | 131.18 | 0.51 |  |  |  |
|  | R012-14-26 | 130 | 25 | 121.34 | 0.21 | 62 | 32; 78 | <0.001 |
|  | Fx012-14-26 | 129 | 37 | 121.73 | 0.30 | 44 | 5; 67 | 0.030 |
| Kenya | Control | 107 | 267 | 92.44 | 2.89 |  |  |  |
|  | R012-14-26 | 115 | 165 | 97.56 | 1.69 | 46 | 25; 61 | <0.001 |
|  | Fx012-14-26 | 122 | 161 | 101.13 | 1.59 | 51 | 31; 65 | <0.001 |
| M20–M32 | |  |  |  |  |  |  |  |
| Ghana | Control | 132 | 52 | 133.28 | 0.39 |  |  |  |
|  | R012-20 | 123 | 20 | 124.60 | 0.16 | 60 | 29; 78 | 0.002 |
|  | Fx017-20-32 | 137 | 29 | 137.36 | 0.21 | 47 | 11; 68 | 0.017 |
| Kenya | Control | 99 | 217 | 82.20 | 2.64 |  |  |  |
|  | R012-20 | 107 | 165 | 89.28 | 1.85 | 34 | 1; 55 | 0.042 |
|  | Fx017-20-32 | 124 | 148 | 105.17 | 1.41 | 48 | 25; 64 | <0.001 |
| M7.5–M26 | |  |  |  |  |  |  |  |
| Ghana | Control | 131 | 90 | 191.38 | 0.47 |  |  |  |
|  | Fx017-20-32 | 141 | 47 | 204.38 | 0.23 | 59 | 30; 76 | 0.001 |
| Kenya | Control | 105 | 358 | 129.92 | 2.76 |  |  |  |
|  | Fx017-20-32 | 133 | 241 | 173.18 | 1.39 | 54 | 38; 65 | <0.001 |
| M2.5–M26 | |  |  |  |  |  |  |  |
| Ghana | Control | 141 | 112 | 254.72 | 0.44 |  |  |  |
|  | R012-20 | 134 | 39 | 240.54 | 0.16 | 65 | 43; 78 | <0.001 |
|  | R012-14-26 | 135 | 47 | 243.61 | 0.19 | 59 | 35; 74 | <0.001 |
|  | Fx012-14-26 | 136 | 72 | 243.30 | 0.30 | 36 | 3; 58 | 0.037 |
| Kenya | Control | 124 | 433 | 190.39 | 2.27 |  |  |  |
|  | R012-20 | 125 | 345 | 198.85 | 1.73 | 27 | 4; 44 | 0.022 |
|  | R012-14-26 | 129 | 276 | 206.71 | 1.34 | 45 | 28; 59 | <0.001 |
|  | Fx012-14-26 | 135 | 285 | 213.57 | 1.33 | 46 | 28; 60 | <0.001 |
| M7.5–M32 | |  |  |  |  |  |  |  |
| Ghana | Control | 131 | 118 | 259.70 | 0.45 |  |  |  |
|  | Fx017-20-32 | 141 | 59 | 278.47 | 0.21 | 59 | 34; 75 | <0.001 |
| Kenya | Control | 105 | 462 | 169.82 | 2.72 |  |  |  |
|  | Fx017-20-32 | 133 | 327 | 227.84 | 1.44 | 52 | 35; 65 | <0.001 |
| M2.5–M32 | | |  |  |  |  |  |  |
| Ghana | Control | 141 | 141 | 326.64 | 0.43 |  |  |  |
|  | R012-20 | 134 | 53 | 305.39 | 0.17 | 63 | 41; 77 | <0.001 |
|  | R012-14-26 | 135 | 53 | 307.79 | 0.17 | 64 | 43; 77 | <0.001 |
|  | Fx012-14-26 | 136 | 82 | 308.51 | 0.27 | 43 | 14; 62 | 0.007 |
| Kenya | Control | 124 | 539 | 233.94 | 2.30 |  |  |  |
|  | R012-20 | 125 | 434 | 243.66 | 1.78 | 26 | 2; 44 | 0.038 |
|  | R012-14-26 | 129 | 330 | 255.01 | 1.29 | 48 | 30; 61 | <0.001 |
|  | Fx012-14-26 | 135 | 337 | 261.01 | 1.29 | 48 | 30; 61 | <0.001 |
| **VE against all clinical malaria episodes meeting the secondary case definition, per-protocol set** | | | | | | | |  |
| M14–M26 | |  |  |  |  |  |  |  |
| Ghana | Control | 136 | 97 | 130.03 | 0.75 |  |  |  |
|  | R012-14-26 | 130 | 36 | 120.95 | 0.30 | 62 | 39; 77 | <0.001 |
|  | Fx012-14-26 | 129 | 54 | 121.09 | 0.45 | 43 | 13; 63 | 0.010 |
| Kenya | Control | 107 | 446 | 85.68 | 5.21 |  |  |  |
|  | R012-14-26 | 115 | 293 | 92.82 | 3.16 | 44 | 25; 58 | <0.001 |
|  | Fx012-14-26 | 122 | 264 | 97.22 | 2.72 | 53 | 37; 65 | <0.001 |
| M20–M32 | | |  |  |  |  |  |  |
| Ghana | Control | 132 | 81 | 132.18 | 0.61 |  |  |  |
|  | R012-20 | 123 | 39 | 123.91 | 0.31 | 53 | 23; 72 | 0.003 |
|  | Fx017-20-32 | 137 | 57 | 136.29 | 0.42 | 36 | 0; 59 | 0.052 |
| Kenya | Control | 99 | 348 | 77.30 | 4.50 |  |  |  |
|  | R012-20 | 107 | 295 | 84.44 | 3.49 | 30 | 0; 51 | 0.048 |
|  | Fx017-20-32 | 124 | 252 | 101.3 | 2.49 | 50 | 29; 64 | <0.001 |
| M7.5–M26 | |  |  |  |  |  |  |  |
| Ghana | Control | 131 | 129 | 189.88 | 0.68 |  |  |  |
|  | Fx017-20-32 | 141 | 72 | 203.42 | 0.35 | 57 | 33; 73 | <0.001 |
| Kenya | Control | 105 | 603 | 120.65 | 5.00 |  |  |  |
|  | Fx017-20-32 | 133 | 429 | 166.05 | 2.58 | 53 | 39; 64 | <0.001 |
| M2.5–M26 | |  |  |  |  |  |  |  |
| Ghana | Control | 141 | 159 | 252.92 | 0.63 |  |  |  |
|  | R012-20 | 134 | 63 | 239.63 | 0.26 | 61 | 42; 74 | <0.001 |
|  | R012-14-26 | 135 | 71 | 242.72 | 0.29 | 58 | 37; 72 | <0.001 |
|  | Fx012-14-26 | 136 | 102 | 242.16 | 0.42 | 38 | 10; 57 | 0.012 |
| Kenya | Control | 124 | 729 | 179.17 | 4.07 |  |  |  |
|  | R012-20 | 125 | 632 | 187.89 | 3.36 | 21 | -2; 38 | 0.072 |
|  | R012-14-26 | 129 | 479 | 199.10 | 2.41 | 46 | 30; 59 | <0.001 |
|  | Fx012-14-26 | 135 | 474 | 206.36 | 2.30 | 48 | 32; 60 | <0.001 |
| M7.5–M32 | |  |  |  |  |  |  |  |
| Ghana | Control | 131 | 173 | 257.60 | 0.67 |  |  |  |
|  | Fx017-20-32 | 141 | 102 | 276.82 | 0.37 | 55 | 31; 70 | <0.001 |
| Kenya | Control | 105 | 760 | 158.55 | 4.79 |  |  |  |
|  | Fx017-20-32 | 133 | 565 | 218.83 | 2.58 | 52 | 36; 64 | <0.001 |
| M2.5–M32 | |  |  |  |  |  |  |  |
| Ghana | Control | 141 | 206 | 324.16 | 0.64 |  |  |  |
|  | R012-20 | 134 | 87 | 304.13 | 0.29 | 60 | 40; 73 | <0.001 |
|  | R012-14-26 | 135 | 84 | 306.63 | 0.27 | 62 | 43; 75 | <0.001 |
|  | Fx012-14-26 | 136 | 122 | 307.01 | 0.40 | 44 | 18; 61 | 0.003 |
| Kenya | Control | 124 | 891 | 220.60 | 4.04 |  |  |  |
|  | R012-20 | 125 | 789 | 230.20 | 3.43 | 19 | -5; 38 | 0.104 |
|  | R012-14-26 | 129 | 575 | 245.66 | 2.34 | 48 | 31; 60 | <0.001 |
|  | Fx012-14-26 | 135 | 561 | 252.55 | 2.22 | 49 | 33; 60 | <0.001 |

M, month; N, number of participants in each group contributing to the considered evaluation period; n, number of episodes included in each group; T (year), person years at risk; n/T, person year rate in each group; VE, vaccine efficacy; CI, confidence interval.

Note: The trial was not powered to assess efficacy by country.

# Supplementary Table 5. Summary of incremental efficacy against all clinical malaria episodes meeting the primary case definition of a fractional third dose administered at month 2, at M26 and M32, overall (exposed set)

| **Time period** | **Group** | **N** | **n** | **T year** | **n/T** | **IVE %** | **95% CI** | **p-value** |
| --- | --- | --- | --- | --- | --- | --- | --- | --- |
|  |  |  |  |  |  |  |  |  |
| M0–M26 | R012-20 | 298 | 429 | 521.15 | 0.82 |  |  |  |
|  | Fx012-14-26 | 304 | 414 | 541.60 | 0.76 | -3 | -33; 20 | 0.816 |
|  | R012-14-26 | 294 | 356 | 527.76 | 0.67 |  |  |  |
|  | Fx012-14-26 | 304 | 414 | 541.60 | 0.76 | -21 | -57; 7 | 0.147 |
| M0–M32 | R012-20 | 298 | 541 | 641.39 | 0.84 |  |  |  |
|  | Fx012-14-26 | 304 | 482 | 668.28 | 0.72 | 6 | -21; 27 | 0.652 |
|  | R012-14-26 | 294 | 422 | 646.75 | 0.65 |  |  |  |
|  | Fx012-14-26 | 304 | 482 | 668.28 | 0.72 | -20 | -56; 7 | 0.163 |

M, month; N, number of participants in each group contributing to the considered evaluation period; n, number of episodes included in each group; T (year), person years at risk; n/T, person year rate in each group; IVE, incremental vaccine efficacy; CI, confidence interval.

# Supplementary Table 6. Cases averted of clinical malaria (secondary case definition) per 1000 children vaccinated and per 1000 RTS,S full-dose equivalents administered, by 3-month periods, from M0 to M32, overall and by country (exposed set)

|  | **Cumulative number of cases averted (95% CI)** | | | | | | | | | | |
| --- | --- | --- | --- | --- | --- | --- | --- | --- | --- | --- | --- |
|  | **Group R012-20** | |  | **Group R012-14-26** | |  | **Group Fx012-14-26** | |  | **Group Fx017-20-32** | |
| **Interval** | **per 1000 children vaccinated*** | **per 1000 full-dose equivalents**** |  | **per 1000 children vaccinated*** | **per 1000 full-dose equivalents**** |  | **per 1000 children vaccinated*** | **per 1000 full-dose equivalents**** |  | **per 1000 children vaccinated*** | **per 1000 full-dose equivalents**** |
| Overall |  |  |  |  |  |  |  |  |  |  |  |
| [0–3] | 56.2  (-51.3; 156.4) | 18.7  (-17.1; 52.1) |  | 121.3  (23.1; 217.4) | 40.4  (7.7; 72.5) |  | 117.8  (22.5; 220.6) | 53.5  (10.2; 100.3) |  | 73.7  (-28.2; 169.6) | 36.9  (-14.1; 84.8) |
| [0–6] | 203.6  (35.4; 356.0) | 67.9  (11.8; 118.7) |  | 309.9  (164.2; 456.6) | 103.3  (54.7; 152.2) |  | 326.3  (182.5; 471.7) | 148.3  (83.0; 214.4) |  | 203.3  (34.7; 352.8) | 101.7  (17.4; 176.4) |
| [0–9] | 335.4  (75.2; 573.8) | 111.8  (25.1; 191.3) |  | 496.6  (280.7; 724.9) | 165.5  (93.6; 241.6) |  | 487.5  (273.9; 709.6) | 221.6  (124.5; 322.5) |  | 355.0  (124.2; 574.7) | 161.4  (56.5; 261.2) |
| [0–12] | 428.6  (59.7; 790.5) | 142.9  (19.9; 263.5) |  | 775.0  (442.2; 1090.8) | 258.3  (147.4; 363.6) |  | 643.4  (329.0; 981.5) | 292.5  (149.5; 446.1) |  | 606.4  (277.1; 941.5) | 275.6  (126.0; 428.0) |
| [0–15] | 516.6  (8.2; 1022.0) | 172.2  (2.7; 340.7) |  | 1054.8  (605.8; 1472.2) | 263.7  (151.5; 368.1) |  | 918.8  (514.5; 1340.1) | 382.8  (214.4; 558.4) |  | 882.4  (428.9; 1320.4) | 401.1  (195.0; 600.2) |
| [0–18] | 641.8  (-3.6; 1265.0) | 213.9  (-1.2; 421.7) |  | 1373.1  (812.3; 1888.4) | 343.3  (203.1; 472.1) |  | 1233.2  (720.2; 1770.9) | 513.8  (300.1; 737.9) |  | 1225.1  (658.7; 1760.7) | 556.9  (299.4; 800.3) |
| [0; 21] | 806.0  (70.1; 1519.0) | 201.5  (17.5; 379.8) |  | 1567.3  (899.1; 2188.7) | 391.8  (224.8; 547.2) |  | 1413.0  (778.9; 2054.4) | 588.8  (324.5; 856.0) |  | 1483.4  (822.6; 2119.7) | 618.1  (342.8; 883.2) |
| [0; 24] | 983.0  (135.3; 1819.2) | 245.8  (33.8; 454.8) |  | 1761.7  (979.9; 2511.3) | 440.4  (245.0; 627.8) |  | 1571.9  (836.5; 2331.1) | 655.0  (348.5; 971.3) |  | 1673.6  (898.7; 2421.0) | 697.3  (374.5; 1008.8) |
| [0; 27] | 1039.6  (83.3; 1981.2) | 259.9  (20.8; 495.3) |  | 1906.4  (1044.0; 2761.1) | 381.3  (208.8; 552.2) |  | 1722.5  (904.0; 2547.3) | 662.5  (347.7; 979.7) |  | 1824.7  (968.8; 2688.4) | 760.3  (403.7; 1120.2) |
| [0; 30] | 1128.8  (42.0; 2168.3) | 282.2  (10.5; 542.1) |  | 2201.6  (1215.3; 3146.3) | 440.3  (243.1; 629.3) |  | 2037.3  (1125.3; 2956.4) | 783.6  (432.8; 1137.1) |  | 1971.9  (1022.9; 2946.9) | 821.6  (426.2; 1227.9) |
| [0; 32] | 1343.5  (220.1; 2438.1) | 335.9  (55.0; 609.5) |  | 2449.9  (1388.0; 3424.4) | 490.0  (277.6; 684.9) |  | 2273.3  (1289.6; 3260.9) | 874.3  (496.0; 1254.2) |  | 2112.1  (1083.3; 3183.7) | 880.3  (451.4; 1326.5) |
| Ghana |  |  |  |  |  |  |  |  |  |  |  |
| [0–3] | 7.9  (-55.3; 71.0) | 2.6  (-18.4; 23.7) |  | 14.5  (-45.8; 74.1) | 4.8  (-15.3; 24.7) |  | -20.0  (-80.3; 43.2) | -9.1  (-36.5; 19.6) |  | 21.6  (-31.6; 74.2) | 10.8  (-15.8; 37.1) |
| [0–6] | 91.6  (-0.8; 180.5) | 30.5  (-0.3; 60.2) |  | 105.5  (9.6; 203.4) | 35.2  (3.2; 67.8) |  | 43.5  (-53.2; 144.3) | 19.8  (-24.2; 65.6) |  | 79.1  (-15.3; 165.3) | 39.6  (-7.7; 82.7) |
| [0–9] | 219.1  (92.9; 353.1) | 73.0  (31.0; 117.7) |  | 177.2  (40.2; 295.3) | 59.1  (13.4; 98.4) |  | 57.1  (-88.9; 208.2) | 26.0  (-40.4; 94.6) |  | 110.3  (-30.0; 252.1) | 50.1  (-13.6; 114.6) |
| [0–12] | 279.0  (110.3; 447.1) | 93.0  (36.8; 149.0) |  | 233.1  (55.1; 400.4) | 77.7  (18.4; 133.5) |  | 53.9  (-142.6; 245.1) | 24.5  (-64.8; 111.4) |  | 197.2  (27.1; 366.2) | 89.6  (12.3; 166.5) |
| [0–15] | 369.6  (179.6; 574.5) | 123.2  (59.9; 191.5) |  | 318.0  (115.6; 520.2) | 79.5  (28.9; 130.1) |  | 146.5  (-73.0; 359.9) | 61.0  (-30.4; 150.0) |  | 305.3  (110.4; 513.8) | 138.8  (50.2; 233.5) |
| [0–18] | 540.4  (313.3; 764.9) | 180.1  (104.4; 255.0) |  | 520.9  (264.3; 754.3) | 130.2  (66.1; 188.6) |  | 282.7  (8.0; 541.9) | 117.8  (3.3; 225.8) |  | 465.9  (194.3; 724.2) | 211.8  (88.3; 329.2) |
| [0; 21] | 605.3  (338.8; 879.2) | 151.3  (84.7; 219.8) |  | 618.0  (334.1; 900.5) | 154.5  (83.5; 225.1) |  | 290.1  (-33.9; 612.6) | 120.9  (-14.1; 255.3) |  | 521.9  (215.0; 834.5) | 217.5  (89.6; 347.7) |
| [0; 24] | 709.4  (422.7; 996.4) | 177.4  (105.7; 249.1) |  | 683.3  (361.6; 981.8) | 170.8  (90.4; 245.5) |  | 335.6  (-31.8; 683.5) | 139.8  (-13.3; 284.8) |  | 523.5  (167.7; 870.3) | 218.1  (69.9; 362.6) |
| [0; 27] | 791.9  (475.9; 1126.7) | 198.0  (119.0; 281.7) |  | 748.7  (402.6; 1082.5) | 149.7  (80.5; 216.5) |  | 365.9  (-52.3; 755.4) | 140.7  (-20.1; 290.5) |  | 608.3  (217.4; 990.1) | 253.5  (90.6; 412.5) |
| [0; 30] | 818.2  (463.4; 1198.7) | 204.6  (115.9; 299.7) |  | 832.7  (422.8; 1206.1) | 166.5  (84.6; 241.2) |  | 435.0  (8.1; 859.5) | 167.3  (3.1; 330.6) |  | 619.2  (192.1; 1038.8) | 258.0  (80.0; 432.8) |
| [0; 32] | 973.2  (571.5; 1368.6) | 243.3  (142.9; 342.2) |  | 1011.2  (587.9; 1448.0) | 202.2  (117.6; 289.6) |  | 574.4  (99.8; 1062.8) | 220.9  (38.4; 408.8) |  | 750.7  (266.9; 1237.1) | 312.8  (112.2; 515.5) |
| Kenya |  |  |  |  |  |  |  |  |  |  |  |
| [0–3] | 112.2  (-85.4; 304.8) | 37.4  (-28.5; 101.6) |  | 239.6  (68.0; 428.7) | 79.9  (22.7; 142.9) |  | 279.7  (112.8; 462.6) | 127.1  (51.3; 210.3) |  | 148.8  (-60.5; 346.6) | 74.4  (-30.3; 173.3) |
| [0–6] | 337.4  (14.0; 634.9) | 112.5  (4.7; 211.6) |  | 542.4  (272.0; 826.4) | 180.8  (90.7; 275.5) |  | 657.7  (388.7; 906.1) | 299.0  (176.7; 411.9) |  | 373.6  (66.7; 656.5) | 186.8  (33.4; 328.3) |
| [0–9] | 483.4  (-12.2; 972.3) | 161.1  (-4.1; 324.1) |  | 871.4  (445.0; 1298.9) | 290.5  (148.3; 433.0) |  | 1008.4  (614.3; 1378.5) | 458.4  (279.2; 626.6) |  | 684.8  (256.8; 1107.2) | 311.3  (116.7; 503.3) |
| [0–12] | 650.1  (-60.0; 1355.2) | 216.7  (-20.0; 451.7) |  | 1474.2  (861.2; 2069.1) | 491.4  (287.1; 689.7) |  | 1432.9  (825.2; 1993.1) | 651.3  (375.1; 906.0) |  | 1214.3  (602.1; 1776.1) | 552.0  (273.7; 807.3) |
| [0–15] | 771.1  (-151.1; 1727.0) | 257.0  (-50.4; 575.7) |  | 2049.0  (1293.6; 2845.6) | 512.3  (323.4; 711.4) |  | 2006.8  (1229.7; 2782.3) | 836.2  (512.4; 1159.3) |  | 1782.2  (932.8; 2552.8) | 810.1  (424.0; 1160.4) |
| [0–18] | 869.3  (-256.6; 1991.8) | 289.8  (-85.5; 663.9) |  | 2563.7  (1664.4; 3579.0) | 640.9  (416.1; 894.8) |  | 2615.2  (1690.6; 3538.5) | 1089.7  (704.4; 1474.4) |  | 2437.4  (1481.2; 3367.8) | 1107.9  (673.3; 1530.8) |
| [0; 21] | 1205.7  (-143.4; 2506.7) | 301.4  (-35.9; 626.7) |  | 2931.0  (1832.1; 4162.3) | 732.8  (458.0; 1040.6) |  | 3072.5  (1910.4; 4149.1) | 1280.2  (796.0; 1728.8) |  | 3020.4  (1865.7; 4124.7) | 1258.5  (777.4; 1718.6) |
| [0; 24] | 1522.3  (-24.6; 3059.1) | 380.6  (-6.2; 764.8) |  | 3359.5  (2043.2; 4854.9) | 839.9  (510.8; 1213.7) |  | 3446.4  (2132.6; 4703.7) | 1436.0  (888.6; 1959.9) |  | 3531.5  (2166.8; 4825.4) | 1471.5  (902.8; 2010.6) |
| [0; 27] | 1557.3  (-171.3; 3256.5) | 389.3  (-42.8; 814.1) |  | 3645.5  (2214.1; 5308.5) | 729.1  (442.8; 1061.7) |  | 3784.7  (2310.0; 5260.9) | 1455.7  (888.5; 2023.4) |  | 3814.6  (2302.3; 5298.7) | 1589.4  (959.3; 2207.8) |
| [0; 30] | 1805.2  (-134.1; 3810.3) | 451.3  (-33.5; 952.6) |  | 4320.0  (2662.3; 6242.2) | 864.0  (532.5; 1248.4) |  | 4524.7  (2814.8; 6259.3) | 1740.3  (1082.6; 2407.4) |  | 4256.0  (2518.1; 5918.7) | 1773.3  (1049.2; 2466.1) |
| [0; 32] | 2483.5  (98.5; 4751.0) | 620.9  (24.6; 1187.8) |  | 5063.1  (2897.8; 7361.2) | 1012.6  (579.6; 1472.2) |  | 5549.7  (3524.4; 7652.6) | 2134.5  (1355.5; 2943.3) |  | 4068.6  (1777.9; 6350.3) | 1695.3  (740.8; 2646.0) |

M, month; CI, confidence interval.

Note: The period M0–M32 was first divided in consecutive 3-month intervals. The number of cases averted per 1000 children vaccinated in each of these 3-month intervals was then computed. The last interval stops at the M32 visit date. The earlier intervals are cut by 3-month intervals (30.5 days times 3). To compute the cumulative number of cases averted, the number of cases averted were summed over all the previous 3-month intervals included in the period considered, starting from day 0. The 95% CIs are the equal-tail quantiles that correspond to the confidence level (2.5% to 97.5%) of the cumulative number of averted cases computed for each of the 1000 resamples with replacement (bootstrapping). For the calculation of cases averted per 1000 RTS,S full-dose equivalents administered, the numbers as calculated above were divided by the total number of full-dose equivalents the children in each group could have received at the end of the previous 3-month interval: for instance, for the total number of cases [0–32]), the full-dose equivalents received at M32 were 4 in the R012-20 group, 5 in the R012-14-26 group, 2.6 in the Fx012-14-26 and 2.4 in the Fx017-20-32 group.

* 95% CIs were computed in a post-hoc analysis.

** post-hoc analysis.

# Supplementary Table 7. Vaccine impact expressed as cumulative number of clinical malaria cases averted (all episodes, secondary case definition) per 1000 RTS,S full-dose equivalents administered when considering that only 4, 3, or 2 fractional doses (0.1 mL) can be withdrawn from a 0.5 mL full-dose vaccine, overall (exposed set)

|  | **Cumulative number of cases averted/1000 full-dose equivalents (95% CI)** | | | | | | |
| --- | --- | --- | --- | --- | --- | --- | --- |
| **Interval** | **Group R012-20** |  | **Group R012-14-26** |  | **Group Fx012-14-26** |  | **Group Fx017-20-32** |
| 4 fractional doses (0.1 mL) are administered from the same vial | | | | | | | |
| [0–] | 18.7 (-17.1; 52.1) |  | 40.4 (7.7; 72.5) |  | 52.4 (10.0; 98.0) |  | 36.9 (-14.1; 84.8) |
| [0–6] | 67.9 (11.8; 118.7) |  | 103.3 (54.7; 152.2) |  | 145.0 (81.1; 209.6) |  | 101.7 (17.4; 176.4) |
| [0–9] | 111.8 (25.1; 191.3) |  | 165.5 (93.6; 241.6) |  | 216.7 (121.7; 315.4) |  | 157.8 (55.2; 255.4) |
| [0–12] | 142.9 (19.9; 263.5) |  | 258.3 (147.4; 363.6) |  | 286.0 (146.2; 436.2) |  | 269.5 (123.2; 418.4) |
| [0–15] | 172.2 (2.7; 340.7) |  | 263.7 (151.5; 368.1) |  | 367.5 (205.8; 536.0) |  | 392.2 (190.6; 586.8) |
| [0–18] | 213.9 (-1.2; 421.7) |  | 343.3 (203.1; 472.1) |  | 493.3 (288.1; 708.4) |  | 544.5 (292.8; 782.5) |
| [0; 21] | 201.5 (17.5; 379.8) |  | 391.8 (224.8; 547.2) |  | 565.2 (311.6; 821.8) |  | 593.4 (329.0; 847.9) |
| [0; 24] | 245.8 (33.8; 454.8) |  | 440.4 (245.0; 627.8) |  | 628.8 (334.6; 932.4) |  | 669.4 (359.5; 968.4) |
| [0; 27] | 259.9 (20.8; 495.3) |  | 381.3 (208.8; 552.2) |  | 626.4 (328.7; 926.3) |  | 729.9 (387.5; 1075.4) |
| [0; 30] | 282.2 (10.5; 542.1) |  | 440.3 (243.1; 629.3) |  | 740.8 (409.2; 1075.1) |  | 788.8 (409.2; 1178.8) |
| [0; 32] | 335.9 (55.0; 609.5) |  | 490.0 (277.6; 684.9) |  | 826.7 (468.9; 1185.8) |  | 844.8 (433.3; 1273.5) |
| 3 fractional doses (0.1 mL) are administered from the same vial | | | | | | | |
| [0–3] | 18.7 (-17.1; 52.1) |  | 40.4 (7.7; 72.5) |  | 50.6 (9.7; 94.7) |  | 36.9 (-14.1; 84.8) |
| [0–6] | 67.9 (11.8; 118.7) |  | 103.3 (54.7; 152.2) |  | 140.0 (78.3; 202.4) |  | 101.7 (17.4; 176.4) |
| [0–9] | 111.8 (25.1; 191.3) |  | 165.5 (93.6; 241.6) |  | 209.2 (117.6; 304.5) |  | 152.4 (53.3; 246.7) |
| [0–12] | 142.9 (19.9; 263.5) |  | 258.3 (147.4; 363.6) |  | 276.1 (141.2; 421.2) |  | 260.3 (118.9; 404.1) |
| [0–15] | 172.2 (2.7; 340.7) |  | 263.7 (151.5; 368.1) |  | 345.4 (193.4; 503.8) |  | 378.7 (184.1; 566.7) |
| [0–18] | 213.9 (-1.2; 421.7) |  | 343.3 (203.1; 472.1) |  | 463.6 (270.8; 665.8) |  | 525.8 (282.7; 755.7) |
| [0; 21] | 201.5 (17.5; 379.8) |  | 391.8 (224.8; 547.2) |  | 531.2 (292.8; 772.3) |  | 557.7 (309.2; 796.9) |
| [0; 24] | 245.8 (33.8; 454.8) |  | 440.4 (245.0; 627.8) |  | 590.9 (314.5; 876.4) |  | 629.2 (337.9; 910.2) |
| [0; 27] | 259.9 (20.8; 495.3) |  | 381.3 (208.8; 552.2) |  | 574.2 (301.3; 849.1) |  | 686.0 (364.2; 1010.7) |
| [0; 30] | 282.2 (10.5; 542.1) |  | 440.3 (243.1; 629.3) |  | 679.1 (375.1; 985.5) |  | 741.3 (384.5; 1107.9) |
| [0; 32] | 335.9 (55.0; 609.5) |  | 490.0 (277.6; 684.9) |  | 757.8 (429.9; 1087.0) |  | 794.0 (407.3; 1196.9) |
| 2 fractional doses (0.1 mL) are administered from the same vial | | | | | | | |
| [0–3] | 18.7 (-17.1; 52.1) |  | 40.4 (7.7; 72.5) |  | 47.1 (9.0; 88.2) |  | 36.9 (-14.1; 84.8) |
| [0–6] | 67.9 (11.8; 118.7) |  | 103.3 (54.7; 152.2) |  | 130.5 (73.0; 188.7) |  | 101.7 (17.4; 176.4) |
| [0–9] | 111.8 (25.1; 191.3) |  | 165.5 (93.6; 241.6) |  | 195.0 (109.6; 283.8) |  | 142.0 (49.7; 229.9) |
| [0–12] | 142.9 (19.9; 263.5) |  | 258.3 (147.4; 363.6) |  | 257.4 (131.6; 392.6) |  | 242.6 (110.8; 376.6) |
| [0–15] | 172.2 (2.7; 340.7) |  | 263.7 (151.5; 368.1) |  | 306.3 (171.5; 446.7) |  | 353.0 (171.6; 528.2) |
| [0–18] | 213.9 (-1.2; 421.7) |  | 343.3 (203.1; 472.1) |  | 411.1 (240.1; 590.3) |  | 490.0 (263.5; 704.3) |
| [0; 21] | 201.5 (17.5; 379.8) |  | 391.8 (224.8; 547.2) |  | 471.0 (259.6; 684.8) |  | 494.5 (274.2; 706.6) |
| [0; 24] | 245.8 (33.8; 454.8) |  | 440.4 (245.0; 627.8) |  | 524.0 (278.8; 777.0) |  | 557.9 (299.6; 807.0) |
| [0; 27] | 259.9 (20.8; 495.3) |  | 381.3 (208.8; 552.2) |  | 492.1 (258.3; 727.8) |  | 608.2 (322.9; 896.1) |
| [0; 30] | 282.2 (10.5; 542.1) |  | 440.3 (243.1; 629.3) |  | 582.1 (321.5; 844.7) |  | 657.3 (341.0; 982.3) |
| [0; 32] | 335.9 (55.0; 609.5) |  | 490.0 (277.6; 684.9) |  | 649.5 (368.5; 931.7) |  | 704.0 (361.1; 1061.2) |

CI, confidence interval.

# Supplementary Table 8. Summary of immune responses to vaccination immunogenicity subset, per-protocol set for immunogenicity

|  | **Group R012-20 (N=46)** | **Group R012-14-26 (N=49)** | **Group Fx012-14-26 (N=45)** | **Group Fx017-20-32 (N=49)** | **Control group (N=48)** |
| --- | --- | --- | --- | --- | --- |
| **Percentage of children with anti-CS antibody concentration ≥1.9 EU/mL, % (95% CI)** | | | | | |
| Pre-vaccination | 4 (1; 15) | 2 (0; 11) | 0 (0; 8) | 4 (0; 14) | 4 (1; 14) |
| 1 month post-dose 2 | 100 (92; 100) | 100 (93; 100) | 100 (92; 100) | 98 (89; 100) | 9 (2; 20) |
| 1 month post-dose 3 | 100 (91; 100) | 100 (92; 100) | 98 (87; 100) | 100 (92; 100) | 5 (1; 15) |
| Pre-dose 4 | 100 (91; 100) | 98 (87; 100) | 100 (91; 100) | 100 (92; 100) |  |
| 1 month post-dose 4 | 100 (90; 100) | 100 (91; 100) | 100 (91; 100) | 100 (91; 100) |  |
| Pre-dose 5 |  | 97 (86; 100) | 100 (90; 100) | 100 (91; 100) |  |
| 1 month post-dose 5 |  | 100 (88; 100) | 100 (87; 100) | 100 (89; 100) |  |
| **Anti-CS antibody GMCs, EU/mL (95% CI)** | | | | | |
| Pre-vaccination | 1.0 (0.9; 1.0) | 1.0 (0.9; 1.0) | 1.0 (1.0; 1.0) | 1.0 (0.9; 1.0) | 1.0 (0.9; 1.1) |
| 1 month post-dose 2 | 376.6 (294.0; 482.4) | 320.0 (248.2; 412.6) | 378.8 (279.3; 513.9) | 253.6 (182.2; 353) | 1.3 (0.9; 1.9) |
| 1 month post-dose 3 | 492.9 (383.8; 633.0) | 342.5 (275.6; 425.6) | 251.1 (170.6; 369.6) | 142.3 (113.9; 177.8) | 1.0 (0.9; 1.1) |
| Pre-dose 4 | 27.7 (19.9; 38.5) | 26.9 (19.2; 37.7) | 22.6 (17.9; 28.5) | 33.3 (23.5; 47.3) |  |
| 1 month post-dose 4 | 288.2 (229.4; 362.1) | 234.8 (184.2; 299.5) | 196.4 (150.4; 256.7) | 187.1 (139.9; 250.1) |  |
| Pre-dose 5 |  | 37.3 (26.7; 52.3) | 38.0 (27.7; 52.1) | 38.9 (27.6; 54.9) |  |
| 1 month post-dose 5 |  | 185.2 (140.4; 244.3) | 145.6 (106.6; 198.9) | 133.9 (103.4; 173.4) |  |
| **Mean anti-CS avidity index* (SD)** | | | | | |
| 1 month post-dose 2 | 37.3 (12.2) | 37.7 (12.1) | 33.4 (13.0) | 36.7 (12.2) | 46.5 (7.7) |
| 1 month post-dose 3 | 37.4 (12.3) | 38.5 (11.1) | 32.9 (11.5) | 39.3 (10.6) | 38.2 (-) |
| Pre-dose 4 | 35.7 (12.8) | 35.8 (10.3) | 29.9 (9.3) | 39.1 (10.1) | 52.4 (-) |
| 1 month post-dose 4 | 42.4 (10.1) | 43.5 (8.3) | 41.4 (11.9) | 44.0 (9.2) |  |
| Pre-dose 5 |  | 34.6 (10.6) | 33.8 (10.4) | 40.6 (8.7) |  |
| 1 month post-dose 5 |  | 37.7 (10.1) | 38 (10.1) | 41.9 (10.9) |  |
| **Percentage of children seroprotected for anti-HBs antibodies (antibody concentration ≥10 mIU/mL), % (95% CI)**** | | | | | |
| Pre-vaccination | 93 (82; 99) | 98 (89; 100) | 89 (75; 96) | 83 (70, 93) | 90 (77; 97) |
| 1 month post-dose 2 | 100 (92; 100) | 100 (93; 100) | 100 (91; 100) | 100 (93; 100) | 91 (80; 98) |
| 1 month post-dose 3 | 100 (90; 100) | 100 (92; 100) | 100 (91; 100) | 100 (92; 100) | 91 (78; 97) |
| Pre-dose 4 | 100 (91; 100) | 100 (91; 100) | 100 (91; 100) | 100 (92; 100) |  |
| 1 month post-dose 4 | 100 (90; 100) | 100 (91; 100) | 100 (91; 100) | 100 (91; 100) |  |
| Pre-dose 5 |  | 100 (91; 100) | 100 (90; 100) | 100 (91; 100) |  |
| 1 month post-dose 5 |  | 100 (88; 100) | 100 (87; 100) | 100 (89; 100) |  |
| **Anti-HBs antibody GMCs, mIU/mL (95% CI)** | | | | | |
| Pre-vaccination | 169.0 (103.0; 277.3) | 224.7 (142.3; 354.7) | 124.6 (72.0; 215.5) | 98.4 (55.7; 173.8) | 152.5 (93.4; 249.0) |
| 1 month post-dose 2 | 41220.9 (24137.4; 70395.3) | 39336.5 (26109.5; 59264.5) | 34301.2 (19896.6; 59134.2) | 29015.8 (17445.6; 48259.4) | 156.7 (83.6; 293.7) |
| 1 month post-dose 3 | 47038.1 (27915.8; 79259.1) | 37592.9 (27463.3; 51458.8) | 21641.4 (12408.6; 37743.9) | 53700.6 (38222.8; 75446.0) | 99.3 (57.4; 171.6) |
| Pre-dose 4 | 5321.5 (3177.2; 8913.0) | 5700.6 (4033.4; 8057.1) | 4856.5 (3129.3; 7537.0) | 9700.1 (6732.0; 13977.0) |  |
| 1 month post-dose 4 | 204372.7 (156739.3; 266481.9) | 137582.9 (108671.7; 174185.8) | 81612.1 (60532.7; 110032.1) | 74185.9 (55296.5; 99528.0) |  |
| Pre-dose 5 |  | 18767.8 (14581.3; 24156.3) | 14130.8 (9985.9; 19996.1) | 13212.6 (9396.3; 18579.0) |  |
| 1 month post-dose 5 |  | 126992.7 (101650.7; 158652.5) | 63683.7 (42424.1; 95596.9) | 57655.7 (44884.3; 74060.9) |  |

N, maximum number of children with available results at any timepoint; CS, circumsporozoite protein; EU, enzyme-linked immunosorbent assay units; CI, confidence interval; GMC, geometric mean concentration; SD, standard deviation; -, not applicable (data for 1 child only); HBs, hepatitis B surface antigen; IU, international units.

Note: *The avidity index was calculated as the ratio of the concentration of anti-CS IgG (EU/mL) that remained bound to the coated antigen after treatment with NH_4_SCN, divided by the concentration of IgG (EU/mL) that remained bound to the coated antigen in the untreated plate.

**Hepatitis B vaccination was an inclusion criterium at enrolment in the study.
